# Deep Learning Models Capture Histological Disease Activity in Crohn’s Disease and Ulcerative Colitis with High Fidelity

**DOI:** 10.1093/ecco-jcc/jjad171

**Published:** 2023-10-10

**Authors:** Dawid Rymarczyk, Weiwei Schultz, Adriana Borowa, Joshua R Friedman, Tomasz Danel, Patrick Branigan, Michał Chałupczak, Anna Bracha, Tomasz Krawiec, Michał Warchoł, Katherine Li, Gert De Hertogh, Bartosz Zieliński, Louis R Ghanem, Aleksandar Stojmirovic

**Affiliations:** AI Lab, Ardigen SA, Kraków, Poland; Faculty of Mathematics and Computer Science, Jagiellonian University, Kraków, Poland; Data Science & Digital Health, Janssen Research & Development, LLC, Spring House, Pennsylvania; AI Lab, Ardigen SA, Kraków, Poland; Faculty of Mathematics and Computer Science, Jagiellonian University, Kraków, Poland; Data Science & Digital Health, Janssen Research & Development, LLC, Spring House, Pennsylvania; AI Lab, Ardigen SA, Kraków, Poland; Faculty of Mathematics and Computer Science, Jagiellonian University, Kraków, Poland; Immunology TA, Janssen Research & Development, LLC, Spring House, Pennsylvania; AI Lab, Ardigen SA, Kraków, Poland; AI Lab, Ardigen SA, Kraków, Poland; AI Lab, Ardigen SA, Kraków, Poland; AI Lab, Ardigen SA, Kraków, Poland; Immunology TA, Janssen Research & Development, LLC, Spring House, Pennsylvania; Department of Pathology, University Hospitals KU Leuven, Belgium; AI Lab, Ardigen SA, Kraków, Poland; Faculty of Mathematics and Computer Science, Jagiellonian University, Kraków, Poland; Immunology TA, Janssen Research & Development, LLC, Spring House, Pennsylvania; Data Science & Digital Health, Janssen Research & Development, LLC, Spring House, Pennsylvania

**Keywords:** Artificial intelligence, inflammatory bowel disease, histology

## Abstract

**Background and Aims:**

Histological disease activity in inflammatory bowel disease [IBD] is associated with clinical outcomes and is an important endpoint in drug development. We developed deep learning models for automating histological assessments in IBD.

**Methods:**

Histology images of intestinal mucosa from phase 2 and phase 3 clinical trials in Crohn’s disease [CD] and ulcerative colitis [UC] were used to train artificial intelligence [AI] models to predict the Global Histology Activity Score [GHAS] for CD and Geboes histopathology score for UC. Three AI methods were compared. AI models were evaluated on held-back testing sets, and model predictions were compared against an expert central reader and five independent pathologists.

**Results:**

The model based on multiple instance learning and the attention mechanism [SA-AbMILP] demonstrated the best performance among competing models. AI-modelled GHAS and Geboes subgrades matched central readings with moderate to substantial agreement, with accuracies ranging from 65% to 89%. Furthermore, the model was able to distinguish the presence and absence of pathology across four selected histological features, with accuracies for colon in both CD and UC ranging from 87% to 94% and for CD ileum ranging from 76% to 83%. For both CD and UC and across anatomical compartments [ileum and colon] in CD, comparable accuracies against central readings were found between the model-assigned scores and scores by an independent set of pathologists.

**Conclusions:**

Deep learning models based upon GHAS and Geboes scoring systems were effective at distinguishing between the presence and absence of IBD microscopic disease activity.

## 1. Introduction

Inflammatory bowel disease [IBD] is a chronic, inflammatory disease of the gastrointestinal tract with symptoms including abdominal pain,diarrhoea, rectal bleeding, and weight loss. It includes two major conditions, ulcerative colitis [UC] and Crohn’s disease [CD]. Recently, clinicians and health authorities have recognised the need to expand from composite clinical disease activity scores to endoscopic and histological assessments in both clinical practice and drug development.^1^ However, despite this increasing emphasis, there is neither consensus in scoring systems nor universally adopted endpoints to measure histological or endoscopic response based on these scores.^2,3^ Therefore, there is a need to develop objective, tissue-based measures of IBD disease activity to improve the assessment of outcomes in IBD clinical trials and definitions of treatment target goals.

The prevailing focus of tissue-based disease activity measures in IBD is on mucosal healing. Whereas endoscopic images enable a gross analysis of the mucosa, histology reflects the microscopic disease processes at specific intestinal locations and is used for the initial diagnosis of IBD. Analyses of clinical trial-derived histology and drug exposure-response data have shown that histological improvement is associated with favourable endoscopic and clinical outcomes across multiple studies.^4,5^ Assessments based on existing histological disease severity scoring systems for clinical trials^6–10^ rely on highly trained human central readers and require substantial time and effort. However, there are shortcomings to this paradigm, including variability inherent to human evaluations and intrinsic limitations of those scoring systems such as low dynamic range and poor sensitivity to meaningful therapeutic effects.

Artificial intelligence [AI] and machine learning [ML] approaches to image analysis are effective and are making great impacts across biomedicine, enabling scaling assessments of disease activity beyond the abilities of human experts.^11,12^ Within histopathology, progress has been most evident in the field of oncology.^13,14^ Many challenges to the applications of AI/ML approaches in clinical practice and clinical trials still remain. These include the requirement for relatively large and well-labelled training datasets,^15^ uneven image quality and specimen collection methods necessitating extensive standardisation and normalisation of data,^16,17^ and hardware limitations encountered when processing large high-resolution images,^18^ as well as ethical and privacy concerns arising from interaction with human subjects.^19^

AI applications in gastroenterology are growing, with most using endoscopy video data. Notable successes have occurred in the detection of intestinal polyps and gastrointestinal malignancy.^20–22^ These approaches benefit from the linkage of endoscopic findings to ground truth provided by well-defined and broadly accepted histological features of cancer and polyps. AI/ML approaches to measure IBD disease activity from endoscopy videos, by comparison, have been more circumscribed and constrained by lacking validated or consensus definitions of mucosal healing. Where developed, the performance of AI/ML algorithms in UC which model existing endoscopic disease activity scoring systems has been comparable to human central readers.^23–28^ In contrast, the application of computer vision/image analysis strategies to IBD histology^29–32^ is less developed and represents a growing opportunity in the field.

We report here the development of AI/ML models that automate the assessment of UC and CD histological disease activity, using a large imaging data archive from Janssen’s late-stage clinical trials.^33–36^ These models were trained on scanned histology slides derived from the intestinal mucosa of adult patients and predict the Global Histology Activity Score [GHAS] for CD^8^ and the Geboes histopathology score for UC.^6^ We found these models to perform well in predicting GHAS and Geboes scores, despite limitations such as imbalanced training data for some GHAS and Geboes subscores and quality variation of histopathology slides.

## 2. Methods

### 2.1. Clinical studies and histological assessments

**Figure 1.**
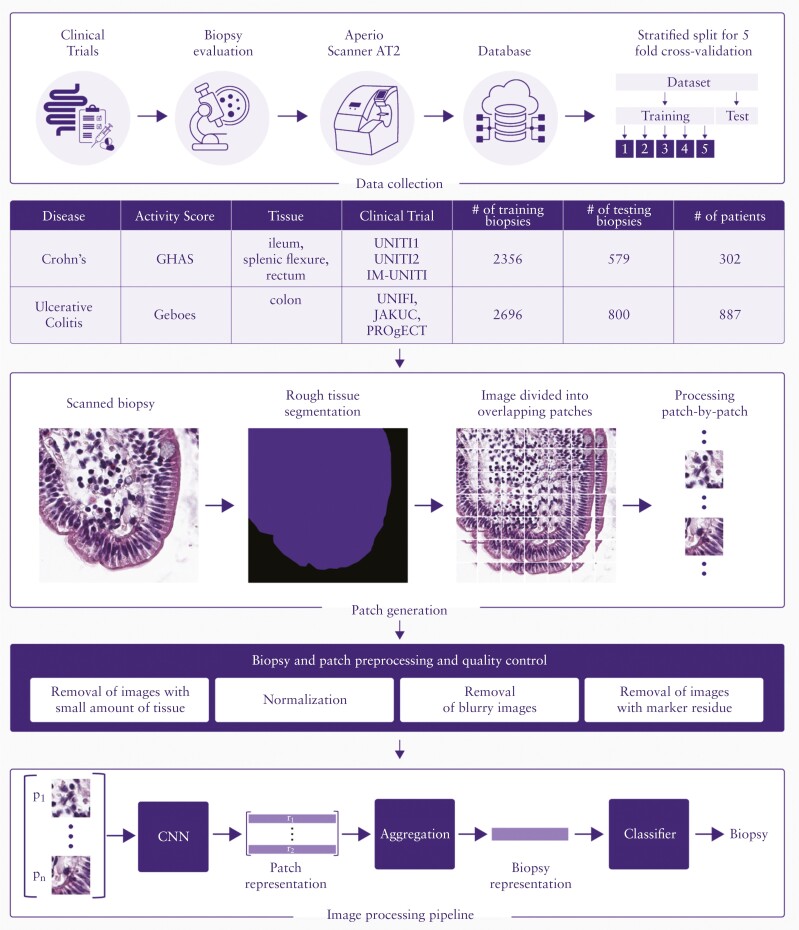
Building the AI pipeline: preparation, preprocessing, model design, and model training. Mucosal biopsies obtained and scored in clinical trials were split into testing and training subsets using a stratified 5-fold cross-validation approach. The distribution and anatomical location of biopsies from IBD trials used in this analysis are shown. Independent models were developed to output GHAS [CD] or Geboes [UC] scores. For GHAS grades, separate models were trained for ileum and colon to account for the differences between their tissue structures. Biopsies from splenic flexure and rectum were merged together for colon model training. Each biopsy image is segmented and split into smaller, overlapping patches that are normalised for staining variability. Patches with quality issues are removed. A summary table describes the biopsy and patch quality review. Finally, a multistage, image-processing workflow was designed to predict each histological score component. First, a Convolutional Neural Network [CNN] is trained on the image patches. Then, a high-dimensional feature vector extracted from the last convolutional layer of the CNN is used as a representation of a patch. Next, the patch representations are aggregated into biopsy-level representations, and the final stage is the classification which assigns the biopsy-level severity scores from biopsy-level representations. AI, artificial intelligence; CD, Crohn’s disease; GHAS, Global Histology Activity Score; IBD, inflammatory bowel disease; UC, ulcerative colitis.

Histological data from six phase 2 and phase 3 clinical trials in CD and UC were used as a reference to develop AI models to predict components of the Global Histological Disease Activity Score [GHAS]^37^ and the Geboes Score^6^ [Figure 1]. The UC studies under consideration were UNIFI^35^[NCT02407236], JAKUC^34^ [NCT01959282], and PROgECT^36^ [NCT01988961], and the CD studies included the three trials collectively referred to as UNITI^33^: UNITI-1 [NCT01369329], UNITI-2 [NCT01369342], and IM-UNITI [NCT01369355]. Briefly, mucosal biopsies from predefined anatomical locations (CD, terminal ileum, splenic flexure, and rectum; UC, rectum and sigmoid colon [the ~15–20 cm from the anal verge that were most representative of disease burden]) as specified by study protocols, were collected in 10% neutral-buffered formalin and processed for haematoxylin and eosin [H&E] staining by a central laboratory. Glass slides were then evaluated and scored in a blinded manner by a single expert gastrointestinal histopathologist to determine the GHAS score in CD and the Geboes Score in UC.

### 2.2. Image dataset

Digital images were generated with an Aperio AT2 scanner at 40x magnification with an average resolution of 10 000 × 10 000 pixels. We collected 2935 biopsies from 302 CD patients and 3496 biopsies from 887 UC patients, across multiple collection time points from IBD clinical trials. Both sets [CD and UC] were then divided into working [80%] and testing [20%] sets with a similar spectrum of histological abnormalities and disease severity.

To train robust models, we applied a 5-fold inner cross-validation method.^38^ For this purpose, we divided the working set in a stratified manner into a training set and validation set, separately for all subgrades and scores. These sets were created after image quality control to obtain a similar spectrum of histological abnormalities and severity across the folds.

### 2.3. Algorithm pipeline

We developed a multi-stage algorithm based on deep learning [Figure 1]. Additional details of the pipeline are in Supplementary Materials.

### 2.4. Evaluation of model performance

Model performance was assessed against central reader scores [full details in Supplementary Materials]. A subset of slides from the UNITI-1 [CD] and PROgECT [UC] studies were used to train an independent panel of five pathologists to assess GHAS and Geboes scores, respectively. All pathologists were diplomates of the American Board of Pathology with a 16-year median length of clinical practice [minimum 10 years, maximum 44 years]. The pathologists were then evaluated on a separate held-back testing set from the same studies. The biopsies in this testing set were also included in the held-back testing set used for AI model evaluation, and therefore could be used to directly compare the performance between AI model assigned scores and those assigned by pathologists. The central reader-assigned scores were taken as the gold standard in these comparisons. The scores assessed by pathologists were summarised by using the modal score for each feature at each anatomical location, to increase the stringency of the comparison. In the event that the mode was not unique, the highest modal score was used. The scores were then converted to simplified binary scores of pathology for analysis. A two-sided Z-test for difference between proportions^39^ at an α = 0.05 was performed to determine whether there were differences between AI model scores and the average of pathologist scores. Inter-rater agreement between the pathologists over the Geboes and GHAS subgrades was assessed by computing Fleiss-kappa statistics.^40^

## 3. Results

### 3.1. The SA-AbMILP model architecture is most suitable for IBD histology severity assessment

To identify the best performing model architecture, we compared three multi-instance learning [MIL] methods—Recurrent Neural Network [RNN], Fisher Vector with Random Forest [FV + RF], and classifier and Self-Attention Attention-based Multi-instance Learning Pooling [SA-AbMILP]—for agreement with the disease severity assignments made by the central reader in both CD and UC. For CD GHAS subgrades in the colon, the SA-AbMILP model exhibited higher accuracy and kappa values in most instances, compared with the FV + RF and RNN models. In the ileum, SA-AbMILP performance was comparable or superior to the FV + RF and RNN models. The SA-AbMILP model, however, demonstrated inferior performance to the FV + RF and RNN models for ulcer detection in both anatomical compartments, having a tendency for false-positive assignments. We observed across all models that agreement with the central reader tended to be better at the extremes of severity [eg, 0 or 3], with lower accuracy for intermediate severities [eg, 1 or 2], across grades.

We also observed that the FV + RF model showed evidence of overfitting to the most represented severity class, for multiple subgrades in both ileum and colon. For this reason, the FV + RF model was not carried forward for assessment of UC or any further analysis. SA-AbMILP model accuracy and kappa values for all UC Modified Geboes subgrades outperformed those of the RNN model. We conclude from these data that the attention model architecture demonstrated the best overall performance for assessing histology across both disease subtypes, and holds the added benefit of interpretability of its output.

### 3.2. The attention model [SA-AbMILP] measures disease pathology with moderate to substantial agreement with the central reader across instruments, anatomy, and conditions [UC, CD]

**Figure 2.**
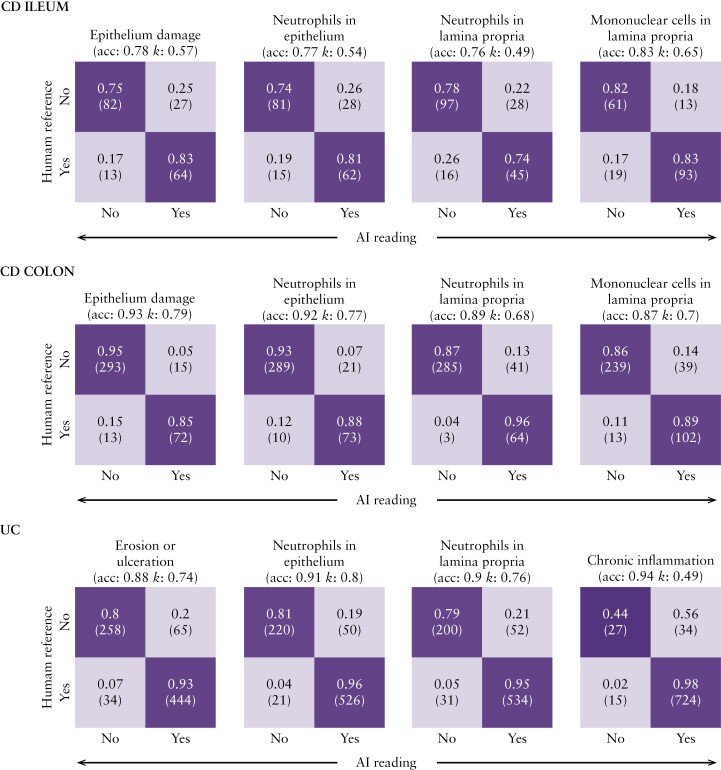
The attention model [SA-AbMILP] can distinguish between the presence and absence of pathology. Confusion matrices are shown for four representative histological features in CD [colon and ileum] and UC [colon]. The features are defined as the presence [YES] versus absence [NO] of pathology and are assessed using the held-back testing set. Each row of the confusion matrix represents the instances assessed by the human reference [central reader], and each column represents the instances predicted by the AI model. The size of each instance class is shown as a proportion of the central reader assessments and, in parentheses, as an absolute count. Accuracy and kappa values are shown for each confusion matrix. The presence of pathology for CD features was derived from GHAS grades as follows. Epithelium damage = EPDAM >0. Neutrophils in epithelium = PolysEP >0. Neutrophils in lamina propria = PolysLP >0. Mononuclear cells in lamina propria = MonosLP >0. The presence of pathology for UC features was derived from Geboes grades as follows. Epithelium damage = Grade 5 >0. Neutrophils in epithelium = Grade 3 >0. Neutrophils in lamina propria = Grade 2B >0. Chronic inflammation = Grade 1 > 0. CD, Crohn’s disease; GHAS, Global Histology Activity Score; UC, ulcerative colitis.

To determine whether the SA-AbMILP model can distinguish between the presence and absence of pathology, we compared model-predicted disease severity against histological readings by the central reader, across four selected histological features, as described in Methods [Figure 2]. We found that model accuracy for colon, in both CD and UC, ranged from 87% to 94% [kappa range: 0.49–0.8] for these features; and that for CD ileum biopsies ranged from 76% to 83% [kappa range: 0.49–0.65]. In addition to this binary analysis of the presence versus absence of pathology, we also evaluated the performance of the SA-AbMILP model over the entire GHAS and Simplified Geboes scales. For GHAS, the accuracy in colon and ileum ranged from 80% to 89% [kappa range: 0.54–0.65], and 65% to 82% [kappa range: 0.46–0.67], respectively. For the Simplified Geboes scale, model accuracy was 65% to 85% [kappa range: 0.44–0.68]. We conclude from these results that the SA-AbMILP model is effective at distinguishing between the presence and absence of pathology, captures disease characteristics across most GHAS and Simplified Geboes grades, and has moderate to substantial agreement with the central reader.

**Figure 3.**
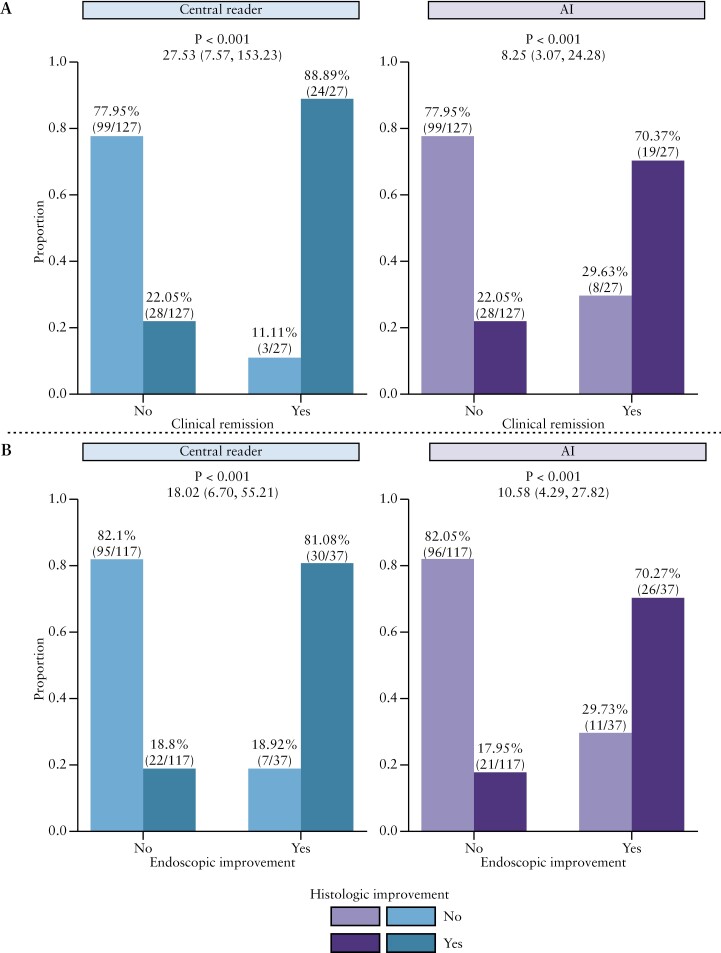
Histological improvement as assessed by the central reader or the attention model [SA-AbMILP] shows similar agreement with clinical remission or endoscopic improvement. Shown are the associations between Week 8 histological improvement [see Methods for definition] and clinical remission [top] and endoscopic improvement [bottom] from UNIFI induction study biopsies. Histological improvement was assessed by the central reader [left, blue colour scheme] and the SA-AbMILP [right, purple colour scheme]. Individual bars in each plot are colour-coded based upon histological improvement [yes or no]. The *p*-value and the odds ratio [with 95% confidence interval] are noted.

It has been established that histological improvement determined by central readers is correlated with clinical remission and endoscopic improvement, following treatment in phase 2 and phase 3 IBD clinical trials.^4,5^ In a subset of our test set, derived from the UNIFI study population of UC patients, we compared the associations between histological improvement with clinical remission and endoscopic improvement according to the central reader’s assessment, and the one obtained by the SA-AbMILP model [Figure 3]. The association with Week 8 clinical remission is significant [all *p* <0.001], whether histological improvement was derived from human central readings (odds ratio [OR] 27.5; 95% confidence interval [CI] 7.6–153) or the SA-AbMILP model [OR 8.3; 95% CI 3.1–24.3], though the former has the greater OR. In a similar vein, endoscopic improvement at Week 8 is significantly associated with histological improvement derived from human central readings [OR 18.0; 95% CI 6.7–55.2] or the SA-AbMILP model [OR 10.6; 95% CI 4.3–27.8]. These data show that the SA-AbMILP model’s predicted histological outcomes demonstrated a significant concordance with clinical and endoscopic outcomes to the same extent as the central reader’s assignments, despite a lower OR.

The model assigned fewer clinical or endoscopic responders as histological responders compared with the central reader (clinical responders: 70% vs 89%; endoscopic responders: 70% vs 81% [model vs central reader]), indicating that the model may have a higher false-positive rate for this classification.

Histological remission in UC trials is often defined as the absence of neutrophils from the colonic mucosa associated with no crypt destruction, and no erosions, ulcerations, or granulation tissue.^41,42^ An important aspect of neutrophil pathology is captured by the Geboes scoring system grades 2B and 3. Although model accuracy for these grades was good [Figure 2], we observed 21% and 19% false-positive rates for the presence of neutrophils based upon GRADE 2B and GRADE 3 assignments, respectively. Conversely, false-negative rates were lower, at 5% and 4% for GRADE 2B and GRADE 3 assignments, respectively. Having observed by confusion matrix analysis that a substantial number of mismatches occurred between adjacent levels of severity for any given grade, we hypothesised that mismatches associated with neutrophil pathology corresponded to not more than a single severity-level difference within a grade. To evaluate this hypothesis, we compared the distributions of disease severity assignments across grades for biopsies where the AI and the central reader assignments disagreed, focusing our analysis on instances of GRADE 2B mismatches. We confirmed that severity level differences within GRADE 2B are almost always within one severity level [eg, 0 vs 1]. Moreover, we found that the disagreement in GRADE 2B tended to be associated with severity-level disagreement for other grades in the Geboes scale as well, except for presence of ulcers, where the agreement was complete between the AI model and the central reader. Across these other grades, severity differences also tended to be limited to a single severity level. Because the SA-AbMILP algorithm allows for interpretability, we selected several examples of disagreements and performed a qualitative analysis with the central reader to determine probable causes for the mismatches. We found that many AI false-positives occurred on slides containing images with staining or other technical artefacts, or histological features such as apoptotic cells [data not shown]. We have also identified cases where neutrophils were accurately captured by the model but not identified by the central reader during the initial scoring assessment. These data indicate that mismatches between the central reader- and AI-assigned scores associated with neutrophil pathology are most often restricted to a single severity-level difference within GRADE2B, but can also be associated with mismatches in other grades.

### 3.3. The SA-AbMILP model has comparable performance to an independent set of pathologists on IBD histology scoring instruments

**Figure 4.**
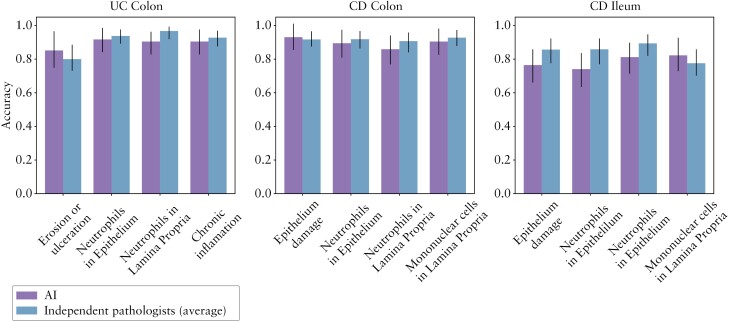
The attention model [SA-AbMILP] showed comparable accuracy to five independent pathologists across most evaluated histological features in CD [colon and ileum] and UC [colon]. The assessment was performed on a held-back testing set, and disease activity for each category was defined as present [YES] vs absent [NO]. Pathologists scores were summarised using the modal score, and the accuracy of this score was evaluated against the central reader. Bar graphs represent accuracy with 95% confidence intervals shown as lines. Two-sided Z-test for difference of proportions, *p <0.05; **, *p* <0.01. CD, Crohn’s disease; UC, ulcerative colitis.

**Figure 5.**
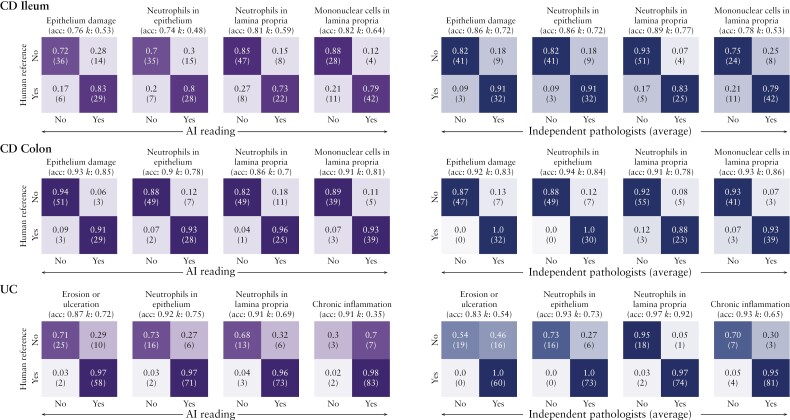
Confusion matrix analysis demonstrates that the attention model [SA-AbMILP] showed comparable accuracy to the modal score of five independent pathologists across four representative histological features in CD [colon and ileum] and UC [colon]. Confusion matrices for the SA-AbMILP compared with the central reader are shown in purple on the left. On the right, shown in blue, are the confusion matrices for the pathologists’ averaged scores compared with the central reader. The assessment was performed on a held-back testing set, and disease severity for each category was defined as present [YES] vs absent [NO]. Each row of the confusion matrix represents the instances assessed by the central reader, and each column represents the instances predicted by the AI model or the average score from five pathologists. The size of each instance class is shown as a proportion of the central reader assessments and, in parentheses, as an absolute count. Accuracy and kappa values are shown for each confusion matrix. AI, artificial intelligence; CD, Crohn’s disease; UC, ulcerative colitis.

To determine whether the model’s performance was comparable to other potential central readers, we next used a portion of the testing set [see ] to compare the relative performance of the SA-AbMILP model against an independent set of experienced pathologists who were trained to use Geboes and GHAS scoring scales [Figure 4, Figure 5], using the original central reader as the gold standard. As above, disease severity was classified as the presence or absence of pathology for four histological features in both UC and CD. To determine the inter-rater agreement between the group of pathologists, we computed agreement statistics using the Fleiss-kappa approach and found moderate agreement between raters for most subgrades. Therefore, to reduce the variation between pathologists and thus increase the stringency of our comparison, the scores assigned by the independent pathologists were summarised for each feature at each anatomical location by using the modal score. For most CD and UC subgrades, we found no significant differences in accuracy between the SA-AbMILP model and pathologists’ modal scores [two-sided Z-test for difference between proportions at α = 0.05]. In two cases, we found a statistically significant difference in favour of the pathologists’ modal scores [neutrophils in epithelium/CD ileum, accuracy 74% [AI] vs 93% [pathologists]; neutrophils in epithelium/CD colon, accuracy 90% [AI] vs 98% [pathologists]. We conclude from these data that the SA-AbMILP model had accuracy comparable to the pooled scores of pathologists for the majority of subgrades.

## 4. Discussion

We developed a collection of AI models to estimate IBD histological disease severity built upon the largest set of H&E-stained biopsy images reported to date. In the initial evaluation of the considered approaches, , the attention model [SA-AbMILP] demonstrated the best performance in terms of accuracy and kappa statistics and was selected for all subsequent modelling of GHAS and modified Geboes subgrades. We found that SA-AbMILP models for each specific subgrade were able to match human central read scores with moderate to substantial agreement. The SA-AbMILP model was effective at distinguishing between the presence and absence of pathology [Figure 2] and comparable to an independent set of experienced pathologists across a variety of GHAS and modified Geboes subgrades [Figure 4, Figure 5]. AI-derived histological disease activity scores were significantly associated with clinical remission and endoscopic improvement in a large clinical trial dataset [UNIFI], similarly to the central read scores [Figure 3].

An important challenge to overcome in building AI models for IBD histology is to first have high-quality images with associated pathology labels. Our dataset consisted of whole-biopsy slide images with an assigned disease severity score comprising several subgrades, without annotation of individual histological features. One approach to model training is to label specific histological features, such as surface epithelium, crypts, lamina propria mononuclear cell infiltrate, or neutrophils, and assess them individually to assign the whole-biopsy disease severity. The output from such models could be readily interpreted by a pathologist. This approach, however, has drawbacks because employing expert gastrointestinal pathologists to label individual histological features in sufficient quantities for use in training deep neural networks is time-consuming, subjective, and is associated with significant costs. To address these drawbacks, we first partition an image into overlapping patches to allow for analysis by convolutional neural networks [CNNs]. Next, to address the challenge of image labelling, we use weakly supervised MIL to learn patch-level estimations from the whole-biopsy labels. Finally, we assemble biopsy-level representations from patches to assess histological severity scores. This aggregation model incorporating an attention mechanism, combined with image preprocessing techniques and methods to address data imbalances, provides a foundation for automating the assessment of IBD histopathology slides.

In our analysis of model performance [Figure 2], we focused on distinguishing between the presence and absence of pathology as a binary outcome. We performed an independent comparison of four measures [intact epithelium, presence of neutrophils in epithelium, presence of neutrophils in the lamina propria, and chronic inflammation] that may characterise the presence and absence of histological inflammation as captured by Geboes and GHAS. Although there remains much debate on which histological scoring instruments should serve as a ‘gold standard’ reference in clinical trials,^2,3^ using binary measures of histological features present in both UC and CD pathology allowed us to compare results across these two conditions. Our approach captures the most relevant factors used in clinical practice which also are highlighted in recent surveys of gastrointestinal pathologists and feature prominently in recent ECCO treat-to-target guidelines.^43^ We found that AI model accuracy for these binary outcomes compared with central-read scores in colon for both UC and CD was greater than 87% for all criteria, and as high as 94% for chronic inflammation in UC. The accuracy for CD ileum across criteria ranged between 76% and 83%. The weaker performance of the models for CD ileum using this binary classification approach may be explained by the fact that fewer training data points were available for this class [CD ileum biopsies: 934, CD colon biopsies: 2011]. We also found for CD subgrades describing neutrophils in the epithelium, that AI model accuracy in our test set compared with independent pathologists was significantly lower in both ileum and colon [Figure 4]. A significant difference, however, was not observed in our UC testing set for these subgrades. This suggests that our model performs well for subgrades where training data volume is higher. Alternative AI model training approaches that use patch-level labels of neutrophils may be necessary to further increase accuracy when training data are limited.

We observed a decrease in model performance when accuracy was evaluated using the full scale for each disease activity measure compared with accuracies derived from the presence or absence of pathology [ie, binary measures—Figure 2]. We speculate that there are several factors that account for these differences. For example, the algorithm may miss cell types that have a low frequency on a given slide, such as neutrophils in areas where there is a dense mononuclear inflammatory infiltrate, or where there is a dark-staining [basophilic] regenerating epithelium. Pathologists, in contrast, can scan slides at higher magnifications [eg, 60x] and visualise the slides in the z-axis to confirm a cell type assessment. Other important factors to consider include insufficient training data, variation in human-assigned scores [ie, variation in the ground truth], and imbalanced data within each histological subgrade.

**Figure 6.**
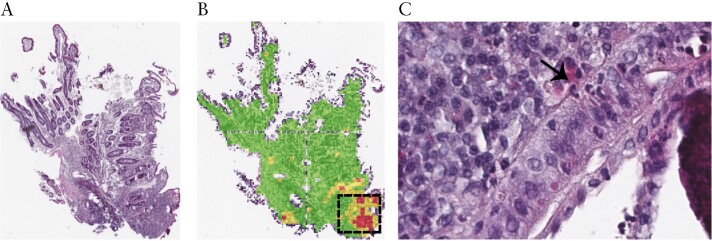
The SA-AbMILP model for disease activity estimation enables the identification of patches within biopsies that contribute to the overall score assignment. Shown is a CD ileum biopsy [A] with a superimposed heatmap of patches associated with neutrophils in the epithelium [GHAS—POLYSEP]. [B] The most relevant patches for the activity assessment are coloured red, intermediate patches coloured yellow, and those less informative coloured green. Examples of H&E-stained neutrophils from the informative region are magnified on the right [C]. CD, Crohn’s disease; GHAS, Global Histology Activity Score; UC, ulcerative colitis.

One advantage of using the attention model architecture is that it provides identification of biopsy regions [patches] that contribute toward score assignment. This enables some interpretability of the AI prediction [Figure 6. During qualitative review of mismatches between the central reader- and AI-assigned scores, we observed many instances where AI model misclassifications occurred on images containing artefacts. The observation that the model assigned fewer clinical or endoscopic responders as histological responders compared with the central reader [Figure 3] is compatible with this interpretation. For pathology attributable to neutrophils in the lamina propria and/or epithelium, our qualitative review suggests the AI model’s performance is sensitive to low neutrophil density. However, we have found instances where apoptotic epithelial cells or a high density of eosinophils in the lamina propria occurred on images that were misclassified by the AI. It is notable that apoptotic cells are often seen in patients under IBD therapy, which may arise during epithelial regeneration where damaged cells present are forced into programmed cell death. The interpretability feature of our model could enable the development of a diagnostic support tool for experienced gastrointestinal [GI] pathologists and, perhaps more importantly, for those pathologists without GI pathology subspeciality expertise. In summary, the advantage of interpretability allowed us to determine that misclassifications are rare and, in some cases, AI models are more sensitive than human readers for important pathology.

We note several limitations to our study. Chief among these is that ground truth GHAS and Geboes scoring assignments were made by a single central reader. To further increase our confidence in the models’ performance, we conducted an association analysis using clinical and endoscopic outcomes as measures independent from the central reader [Figure 3]. We found that model predictions significantly associated with these variables, suggesting that the ground truth assignments were reliable. Although desirable, we did not have GHAS or Geboes assessments from multiple pathologists except for a portion of the testing set. Thus, we used this subset to assess pathologist inter-reader agreement between an independent group of pathologists and to help contextualise AI accuracy against alternative human readers. Inter-reader variation presents a general challenge when attempting to define ground truth.^44^ In our cohort of independent pathologists, we found moderate agreement between readers for the majority of subgrades across instruments. One interpretation is that the scoring instruments as constructed may have intrinsic properties that constrain agreement between experienced pathologists in practice. Our AI model accuracy would be expected to benefit from additional training data derived from ground truth scores provided by groups of experts with high levels of inter-reader agreement.

During the development of our AI systems, we encountered various quality issues resulting in rejection of a substantial proportion of images or individual patches [Figure 1]. We therefore needed to remove these images from the training and testing procedures of the models to avoid misleading the neural network and affecting its final performance [see Supplementary Materials]. We observed that more images were rejected from the UC dataset compared with the CD dataset. This prompted us to further investigate potential causes that could explain this difference. We identified tissue preparations as a root cause. These included differences in tissue thickness, often accompanied by tissue shearing of UC samples, and differences in H&E staining. We also identified temporal batch effects that could be explained by a change in the laboratories processing samples for the UNIFI study. Batch effects associated with laboratory processing, however, did not affect the model’s ability to assign the presence or absence of pathological features for biopsies of sufficient quality. While these quality issues did not affect human central reading, it is possible they impacted on AI-assigned scores and therefore will require further workflow optimisation to account for heterogeneity of tissue preparation. A potential mitigation that would foster future refinement of the AI models is to establish universal guidelines for tissue preparation in IBD clinical trials, including recommendations for optimal paraffin block sectioning. Differences in staining could be mitigated using image processing techniques.^45^

An important and common challenge for AI modelling is the prospect of unbalanced data. In our study, we observed that the distribution of severity score values for GHAS and Geboes subgrades suffers from class imbalances. Interestingly, we observed bidirectional imbalances. For example, some subgrades are skewed towards normal histology [GHAS for colon—polysLP, polysEP, ulcer] and other subgrades are skewed towards severe histology [Geboes—grade 0 and grade 1]. In CD, the observed imbalances differed between colon and ileum. In colon, all GHAS subgrades were skewed towards normal. In contrast imbalances in the ileum, when present, were less pronounced and were skewed towards severe in subgrades corresponding to chronic inflammation. We speculate that this observation stems from both the patchy nature of inflammation in CD and the fact that enrolment in these clinical trials was not stratified by histological severity. Imbalances such as these are concerning because they may lead to overfitting and hence limit generalisability of ML models. In such cases, accuracy alone is not a valid measure of model performance and can be misleading.

To address the imbalances, we considered a variety of approaches. First, we used confusion matrices and kappa statistics to measure the performance of our models, focusing not only on the overall accuracy across GHAS and Geboes grades, but also on the model’s ability to assess less prevalent subgrade values. Because performance of all models was suboptimal across Geboes scale subgrades, we also simplified the Geboes scale by merging the highest, and usually least represented, values within a subgrade [see Methods]. We considered this refinement acceptable because the distinction between these merged subgrades is often subtle and may be difficult for pathologists to discriminate. In support of this view, pathologists in our study demonstrated slight-to-fair agreement across several subgrades for intermediate levels of severity. Accuracy of the SA-AbMILP model on GHAS grades was considered acceptable and therefore the GHAS did not require modification. We also modified our training process by using a FocalLoss^46^ function that identifies difficult data points from the model perspective, and increases the penalty for making incorrect predictions related to these data points. The collective impact of these approaches was to improve performance, but additional training data will be needed to further improve our models, most notably for models of CD ileum histology.

Using more than 5000 training biopsy images from over 1000 research subjects, we have developed a collection of IBD histopathology computer vision models that automate the assessment of UC and CD histological disease activity in both ileum and colon. Having a large dataset results in a lower likelihood of models overfitting data, captures a broader range of pathology, and allows for an MIL strategy which spares costly and time-consuming re-annotation of histology slides. These models demonstrate moderate to substantial agreement with the human central reader, and performance comparable to an additional five independent pathologists. Beyond using additional training data, opportunities to improve model performance in the future include employing more recent computer vision approaches, such as vision transformers trained in a self-supervised manner,^47^ or using labelled histological features [image segmentation]. The automation of existing histological disease severity measures may improve accuracy, reduce variability, and increase access to reliable IBD histological grading where an expert GI pathologist is not available in clinical practice.

Another application of AI/ML approaches would be to develop novel disease activity measures from image features, which may be more sensitive to clinical characteristics or treatment outcomes. These could include a measure of neutrophil abundance, or models trained on biomarkers such as serum proteins or tissue gene expression. Combining similar outcome measures [eg, neutrophils in the epithelium] that are common across instruments for UC and CD, and therefore making use of larger data pools for model training,, may allow for the integration of IBD subtypes [CD and UC] into one instrument for each anatomical location. Such novel endpoints would require additional, well-characterised, training image data and subsequent validation in an independent cohort.

In summary, advances in histological assessment by AI/ML methods offer the promise of improving the assessment of outcomes in IBD clinical trials, and may identify new treatment target goals leading to better long-term outcomes for patients.

## Supplementary Material

jjad171_suppl_Supplementary_Material

jjad171_suppl_Supplementary_Table_S2

## Data Availability

Study data will not be shared.
