# Supplementary material for: Deep Learning Models Capture Histological Disease Activity in Crohn’s Disease and Ulcerative Colitis with High Fidelity
Source: J Crohns Colitis. 2023 Oct 10;18(4):604–14. doi: 10.1093/ecco-jcc/jjad171 (PMC11037111; doi:10.1093/ecco-jcc/jjad171)
Supplement: jjad171_suppl_Supplementary_Material [file jjad171_suppl_supplementary_material.docx]

**Title**

Deep learning models capture histological disease activity in Crohn’s Disease and Ulcerative Colitis with high fidelity

**Supplementary Materials**

**Histologic Assessments**

The GHAS was initially developed to assess histologic abnormalities in CD [^1^](#_ENREF_1). The score includes features of epithelial damage as well as acute and chronic inflammatory changes. A modified version of the original GHAS, excluding the presence of granulomas and the component of number of affected biopsies, was used in UNITI studies [^2^](#_ENREF_2). Adjacent biopsy pairs collected from the rectum, splenic flexure and terminal ileum (up to six biopsies per slide) were scored. Descriptions of the individual categories and associated grades are presented in Fig. S1.

The Geboes grading scale for histological assessment of inflammation in UC was developed to assess progressively more severe inflammation and disease activity based on histologic criteria [^3^](#_ENREF_3). Descriptions of the individual categories and associated grades are presented in Fig. S2. Due to the high imbalance between data classes in the UC data (see Fig. S3), we simplified the Geboes scores. In most cases, we merged the two highest, usually least represented, severity values (Fig. S4).

**Binary Histopathology Measures Derived from GHAS and Geboes Scoring Systems**

Four histological features were selected for development of binary outcome measures: Epithelial damage / ulcerations and erosions (GHAS EPDAM, Geboes GRADE5), Neutrophils in epithelium (GHAS POLYSEP, Geboes GRADE3), Neutrophils in lamina propria (GHAS POLYSLP, Geboes GRADE2B); Mononuclear cells in lamina propria / chronic inflammation (GHAS MONOSLP, Geboes GRADE1). For each category, the absence of pathology was represented by GHAS or Geboes scores of zero and the presence of pathology was represented by non-zero scores.

**Algorithm Pipeline**

**Preprocessing, Generating Patches and Quality Control**

Several strategies were employed for image processing and quality control. For CD, whole slide images containing more than one biopsy section (up to 6) were digitally annotated to identify individual biopsy sections and associate each section with the appropriate anatomical region (ileum, splenic flexure and rectum). The same approach was utilized for UC without anatomical annotation because all biopsies were obtained from the recto-sigmoid colon. Portions of the slide image without biopsy tissue (background) were removed by tissue segmentation using the Otsu method[^4^](#_ENREF_4). A sliding window mechanism limited to the segmented area was used to split the image into patches of resolution 512 x 512 pixels with an overlap of 256 pixels. The patch representation model architecture requires that patch size and microscopy magnification be fixed. For each independent patch, staining was standardized using self-attentive adversarial staining normalization [^5^](#_ENREF_5).

Image quality control was performed to remove blurry patches, background patches, and patches with a green marker residue because such patches could be detrimental to model training and have the potential to bias model output (Fig. S12). Blurry patches were removed using the SVM classifier [^6^](#_ENREF_6) trained on the manually selected blurry and clear patches represented by a histogram of oriented gradients[^7^](#_ENREF_7). To detect background patches, images were transformed into HSV (Hue, Saturation, Value) color space. Patches having the 5th percentile of the saturation channel smaller than 0.01 (value obtained empirically), corresponding mostly to white space, were removed. A green marker was used on slides to mark individual biopsies, leaving a residue that, in some instances, overlapped the biopsy image. To remove this artifact, a mean channel intensity was first calculated for each RGB channel in the image. If the green channel mean was higher than the blue or red channels, the entire patch was removed.

**Generating Patch Representations**

ResNet18[^8^](#_ENREF_8), an artificial neural network, was used to develop a patch representation model. ImageNet[^9^](#_ENREF_9), a large database of natural images, was used in a transfer learning approach to generate a pre-trained model in order to overcome the relatively small number of biopsy training images with appropriate labels. The last layer of this pretrained model was replaced with neurons corresponding to histology severity scores (GHAS or Geboes). The network was then fine-tuned to predict the biopsy-level scores for all patches comprising a biopsy image. While such classification is imperfect, it is acceptable because the resulting network is used exclusively to generate patch representations [^10^](#_ENREF_10), taken as the activations of the penultimate layer.

The patch representation network was trained until it converged, using an early stopping mechanism. The following parameters were used: batch size 64, Adam optimizer [^11^](#_ENREF_11) with β_1_ = 0.9 and β_2_ = 0.999, and MSE loss with learning rate 0.001. Additionally, exhaustive data augmentation was performed using rotation, skewing, flipping, and shearing with the probability of 0.5 for each operation.

Training a network to predict histology severity scores presents a challenge because each scoring system, GHAS or Geboes, has multiple subscores that would require the development of numerous individual models, one for each subscore. Many subscores were observed to be correlated (Fig. S15). Therefore, to simplify patch representation training and increase model accuracy, histologically similar or correlated subscores were grouped within each scoring framework (GHAS or Geboes, Supp. Table 1) and a single network was trained for each group using multitask learning[^12^](#_ENREF_12).

Supplementary Table 1: grouping for patch representation model training:

|  | GHAS | Geboes |
| --- | --- | --- |
| Group 1: | POLYSLP, POLYSEP, EPDAM | GRADE2A, GRADE2B, GRADE3 |
| Group 2: | ARCHCH, MONOSLP | GRADE0, GRADE1 |
| Group 3: | ULCER | GRADE4, GRADE5 |

**Aggregating Patch Representations and Classifying Histology Scores**

The patch representation model generates representations for all patches comprising a given biopsy image. These representations must be aggregated before prediction of biopsy level histology scores because patch number varies across images. For aggregation, three different methods were used (Fig. S16): Fisher vector and random forest (FV+RF) ^13^, recurrent neural network (RNN) [^14^](#_ENREF_14), or the self-attention attention-based multiple instance learning (SA-AbMILP) model [^15^](#_ENREF_15).

For all methods, we performed a grid search to establish the best hyperparameters. We used: sample_number=10000, cluster_number in range [5; 30] for FV representation and criterion: gini, class_weight: balanced, max_depth in [10; 15], max_features in [10, 15], min_sample_split in [5, 10], n_estimators [25; 75] for RF. For FV we used implementation from cyvlfeat^^[[1]](#footnote-2)^^, while for RF we used scikit-learn^^[[2]](#footnote-3)^^. In the case of RNN we used the following parameters: recurrent_module: GRU, layer_num in [1; 3], output_dim = 512, dropout: 0.0. For the SA-AbMILP module we used: number_of_heads in [1; 3], inner_dim in [128, 256, 512], without kernels. RNN and SA-AbMILP are implemented using PyTorch library^[[3]](#footnote-4)^.

A weighting strategy for all methods was used during training to balance the sub-scores in each category. For attention and Recurrent Neural Networks (RNN) the following parameters were used: focal loss [^16^](#_ENREF_16) or LDAM loss [^17^](#_ENREF_17), which are more effective in dealing with imbalance than a straightforward balancing strategy, early stopping with the window of 25 epochs, and Adam optimizer [^11^](#_ENREF_11) with a learning rate of 0.0001. In addition, these models used a batch size equal to 1, a shallow neural network with two linear layers, ReLU activations as a classifier, and dropout with probability 0.3 before the first classifier layer. Finally, the attention model used a normalizing layer [^18^](#_ENREF_18).

Aggregated biopsy-level representations generated by each model were embedded into a 2D plot using t-SNE algorithm [^19^](#_ENREF_19) to explore relationships between these representations.

**Evaluation of Model Performance**

For the evaluation of model performance, multi-stage image processing models for each subscore were retrained on the entire training set using the best parameters found with cross-validation and were evaluated on the held back testing set. We evaluated these models by assessing their performance on predicting histological subgrades (i.e., Simplified Geboes for UC and GHAS for CD), as well as by comparing their predictions of simplified disease severity measures that were obtained by compressing selected histological subgrades into binary scores (zero vs non-zero values). The binary classification was done to enable comparison between Geboes and GHAS instruments for selected subgrades that have similar but not identical histological definitions, as well as to enhance interpretation by focusing on the measures most relevant in clinical practice and used as endpoints in IBD clinical trials (e.g., presence or absence of neutrophils). Four histological features were selected for development of binary outcome measures (see Supplementary Methods). The disease severity score assignments by AI models were compared to the central reader’s scores and the results were presented as confusion matrices together with total accuracy (proportion of correctly assigned scores) and Cohen’s kappa values [^20^](#_ENREF_20). Disagreements between score assignments by the AI model and the central reader for the presence of neutrophils in the lamina propria were further investigated by a qualitative examination of disease severity distributions across Geboes grades. Several examples of such disagreements were selected for a further qualitative analysis with the central reader.

#### **Clinical Endpoint Associations**

The associations between dichotomous clinical endpoints (clinical remission, endoscopic improvement) and histologic improvement in the UNIFI study [^21^](#_ENREF_21), as assessed by the central reader and the SA-AbMILP model, were evaluated by Fisher’s exact test. Statistical significance was based on nominal p-values at the 0.05 level. Only testing set biopsies from randomized subjects were used for this analysis. Histologic improvement at week 8 in the UNIFI study was based upon the Geboes scale and defined as neutrophil infiltration in < 5% of crypts (maximum GRADE 3.1), no crypt destruction (GRADE 4.0), and no erosions, ulcerations or granulation tissue (GRADE 5.0). Clinical remission was defined as a Mayo score [^22^](#_ENREF_22) not greater than 2 points, with no individual score greater than 1. Endoscopic improvement was defined as achieving a Mayo endoscopic subscore (MES) [^22^](#_ENREF_22) of 0 or 1 (MES ranges from 0 to 3 and the UNIFI subjects had a 2 or 3 at screening).

**Supplementary Figures**


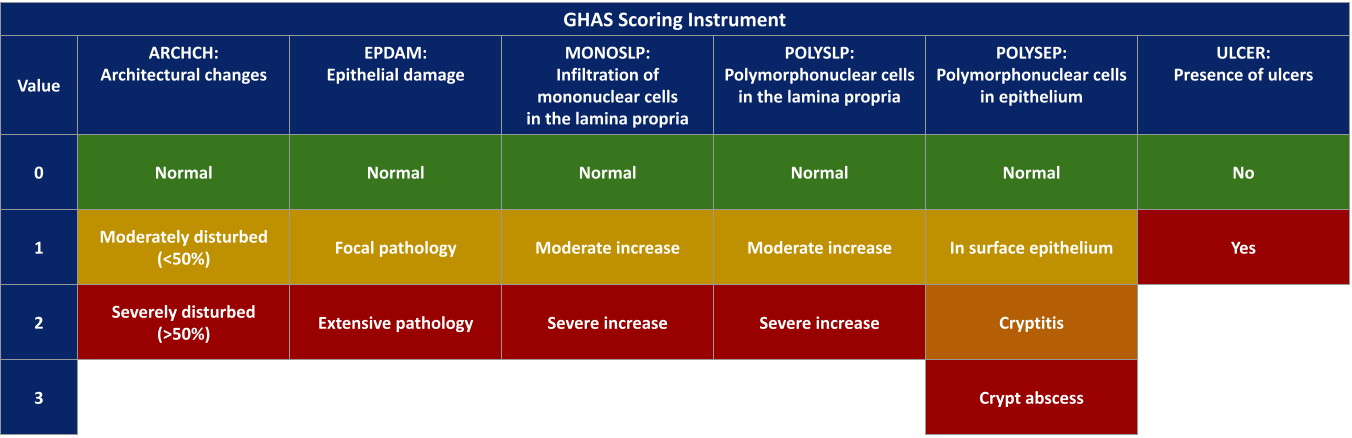


**Fig. S1**


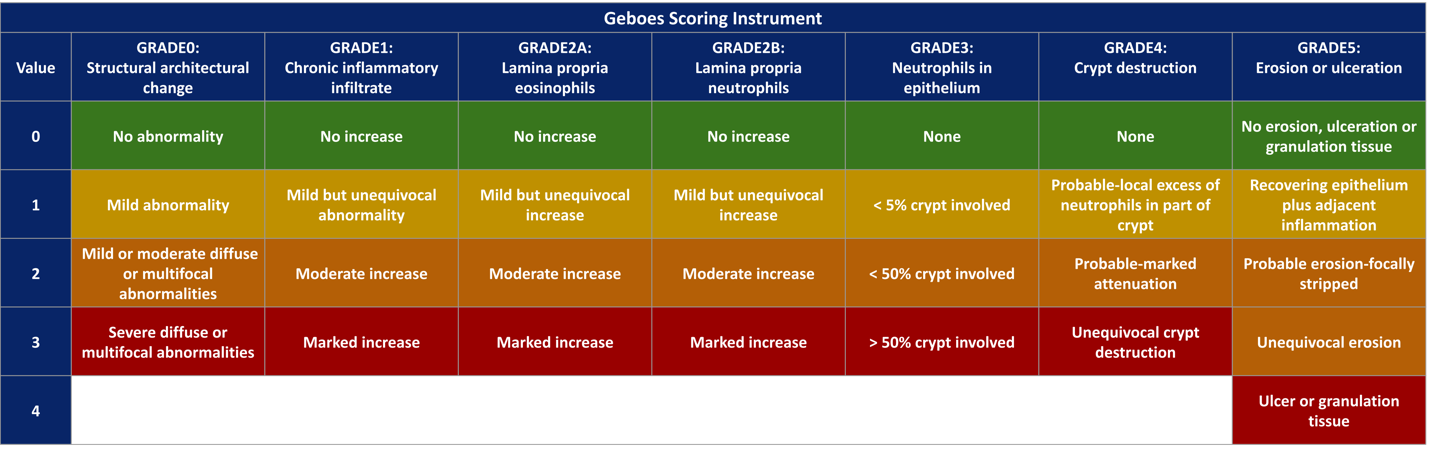


**Fig. S2**

**
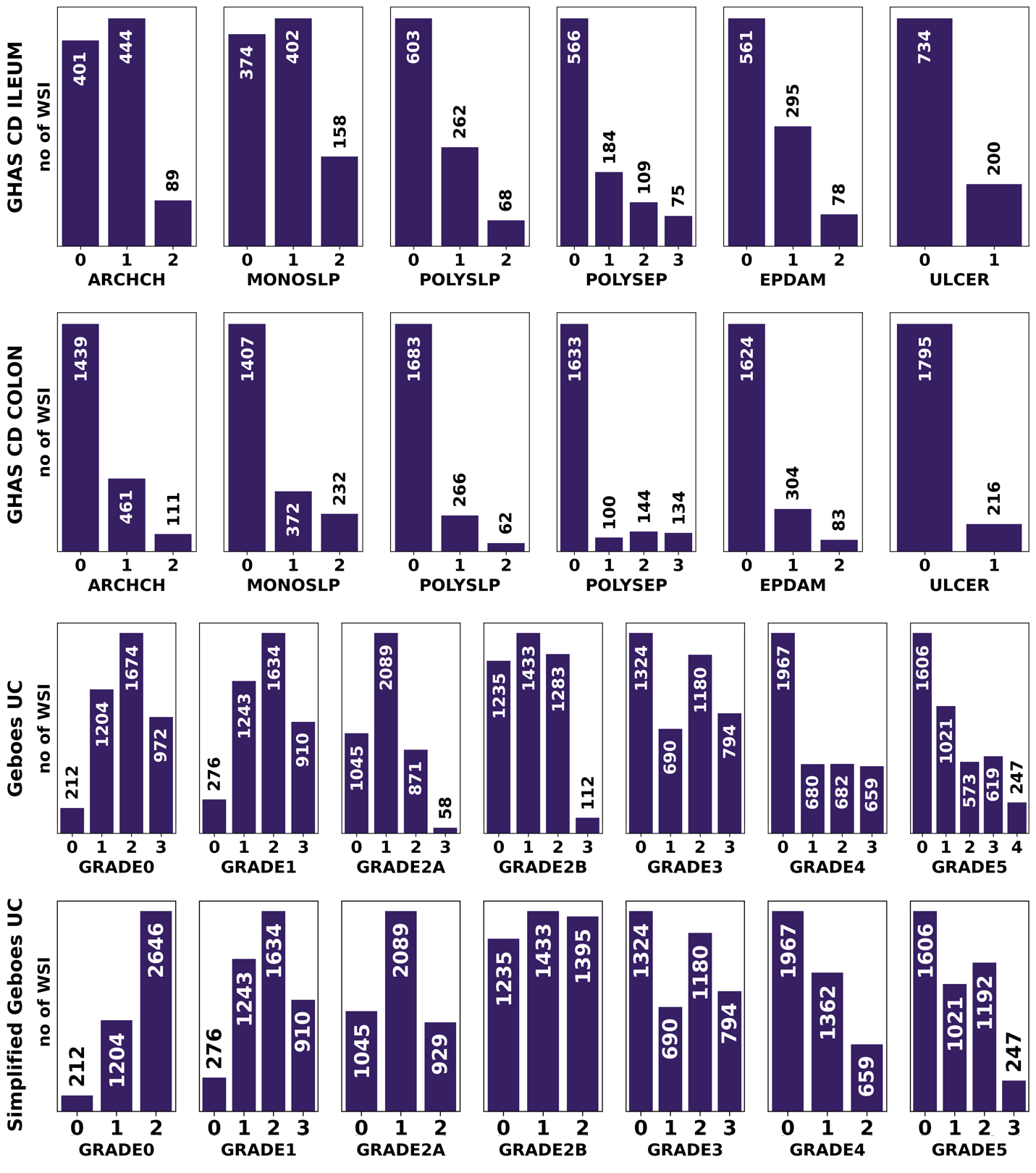

Fig. S3**

**
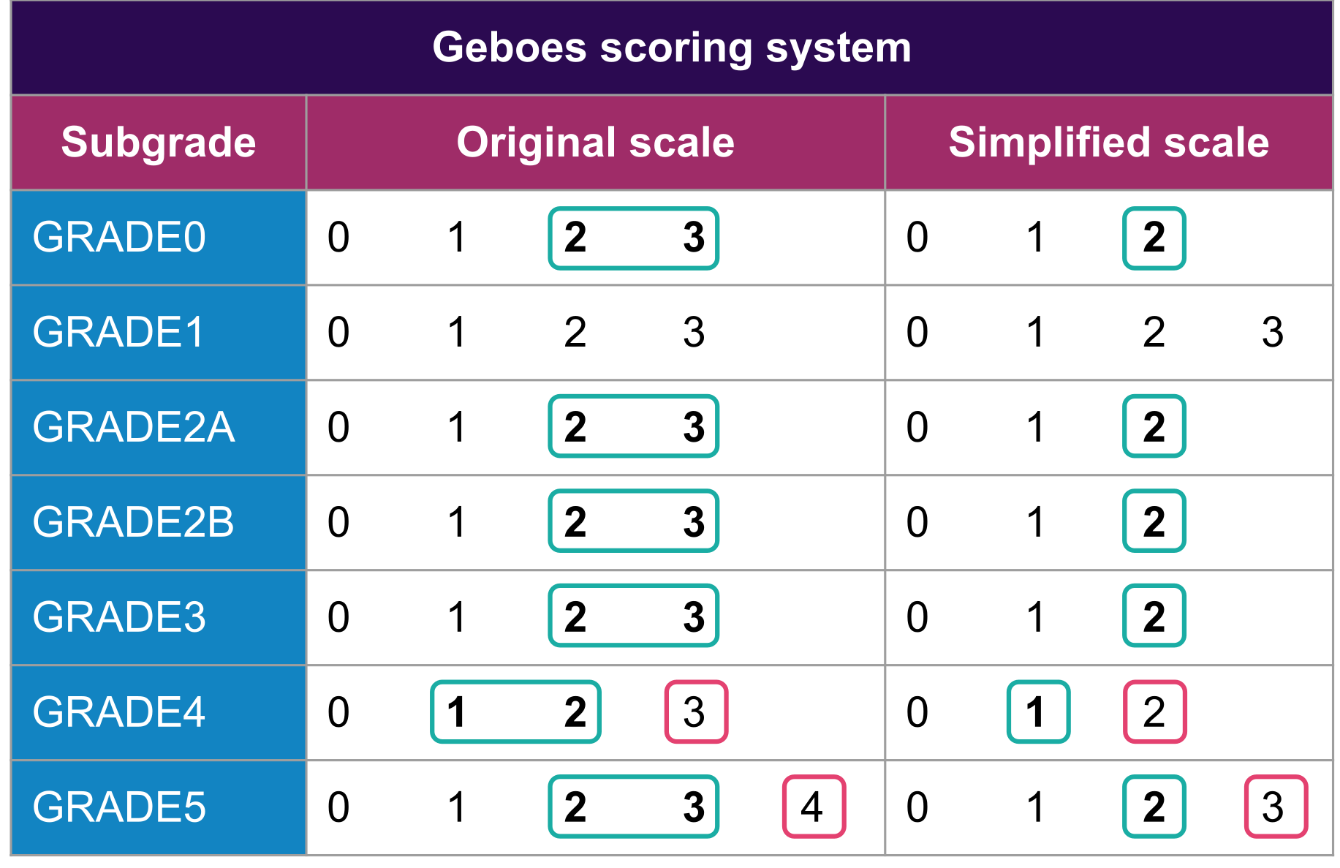
**

**Fig. S4**


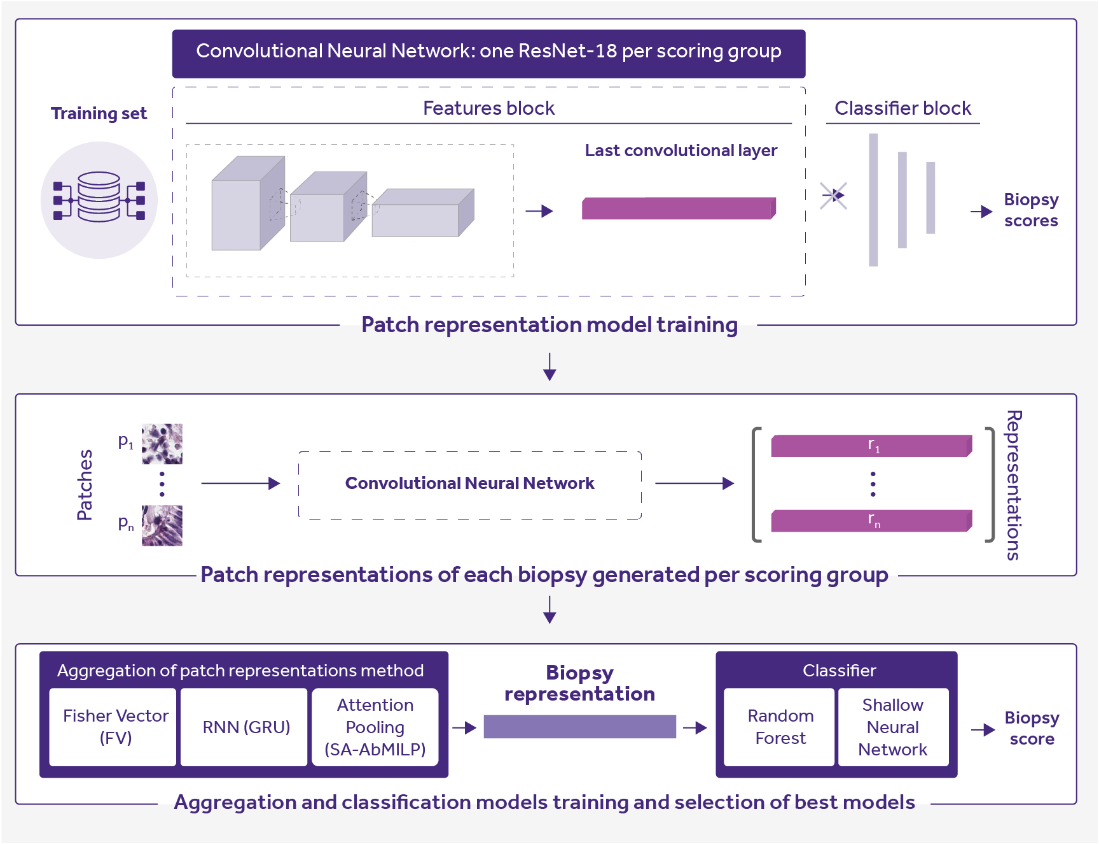


**Fig. S5**


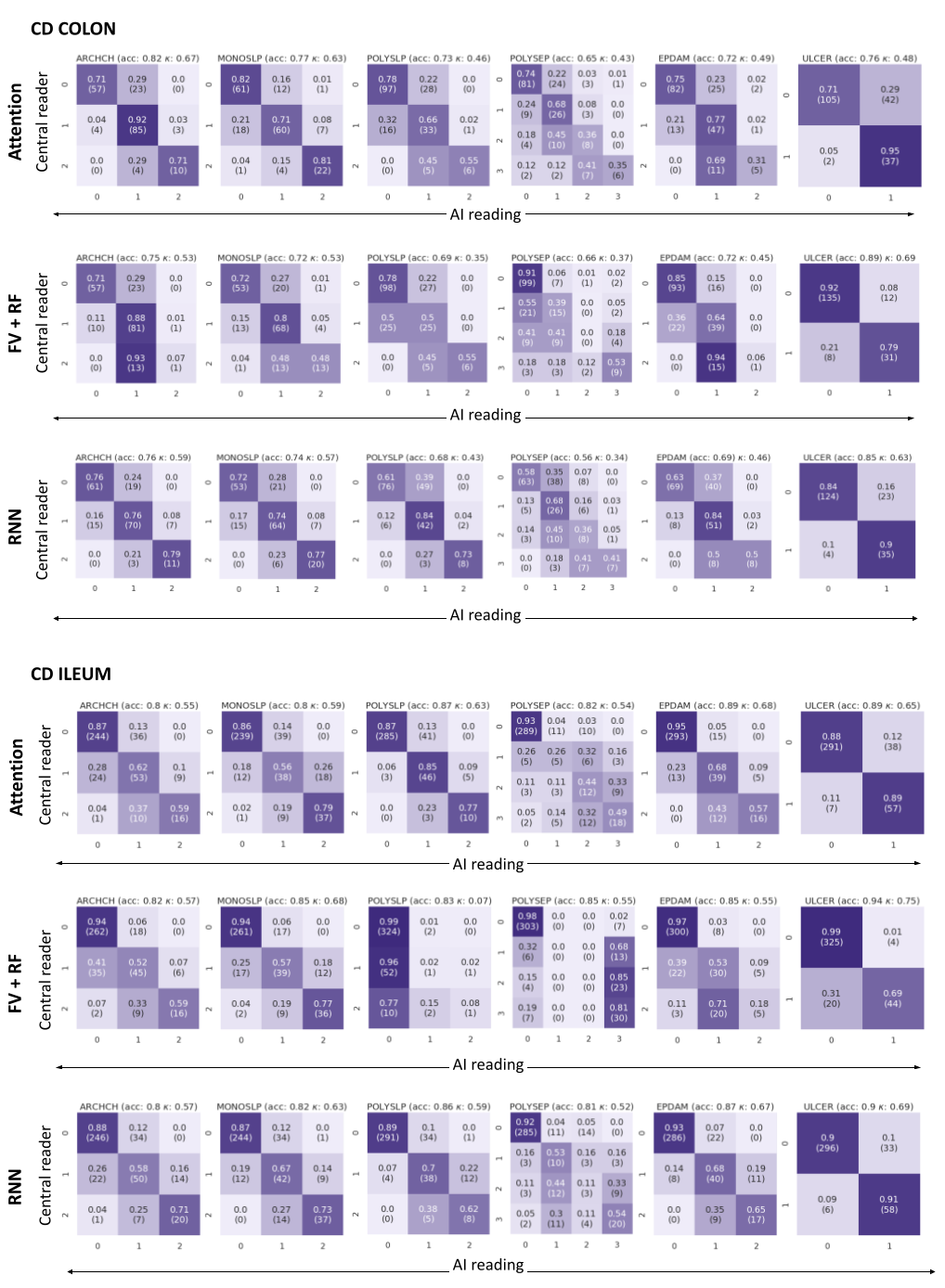


**Fig. S6**


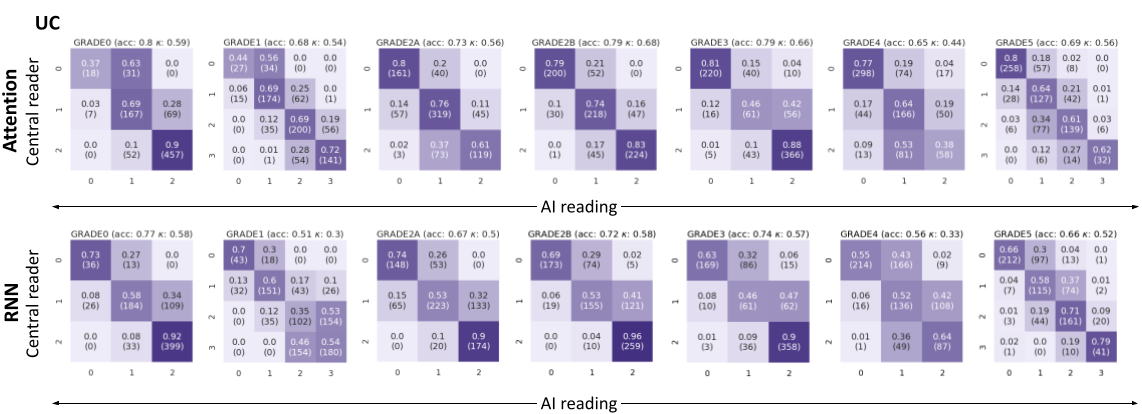


**Fig. S7**

**
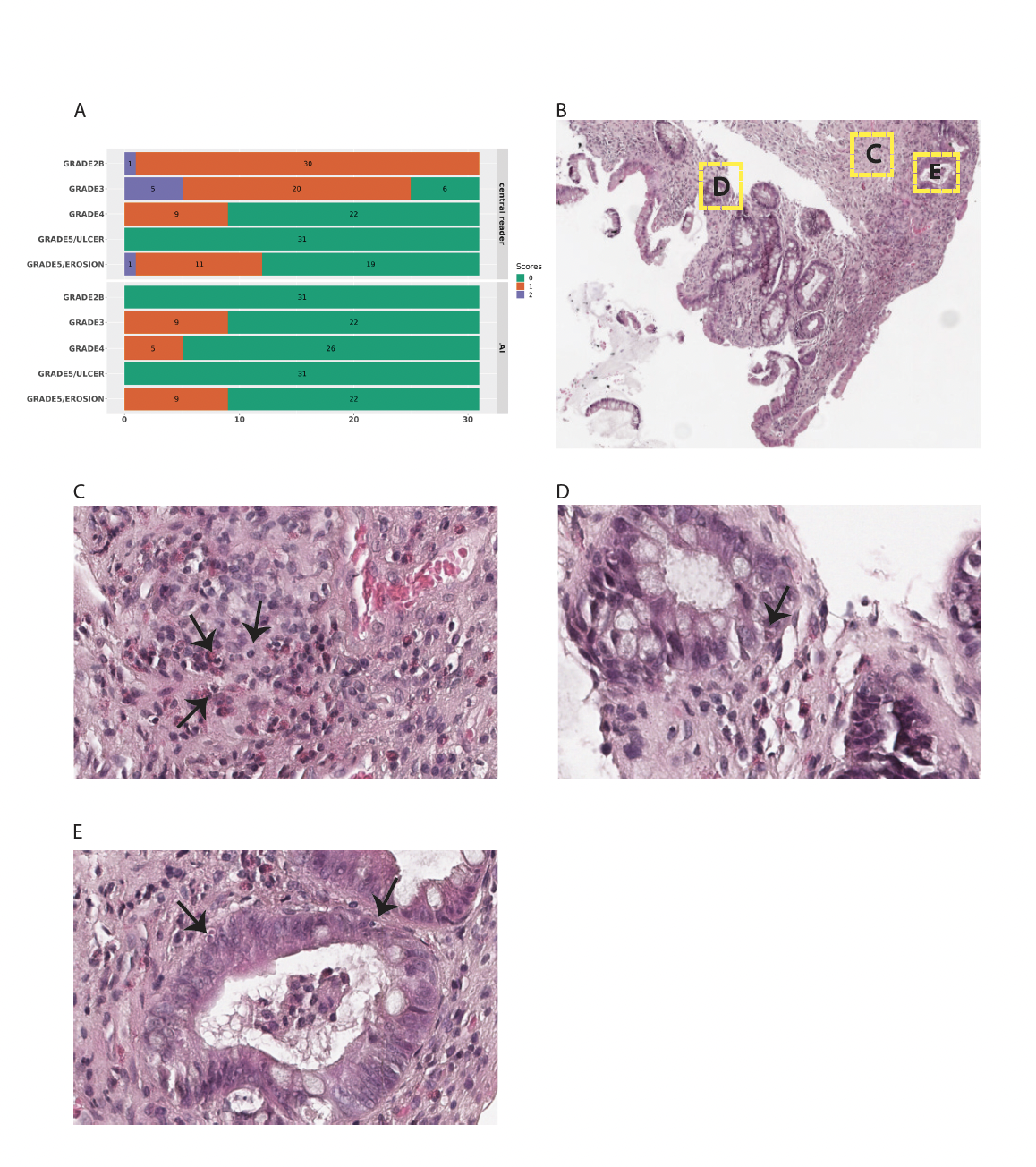
**

**Fig. S8**

**
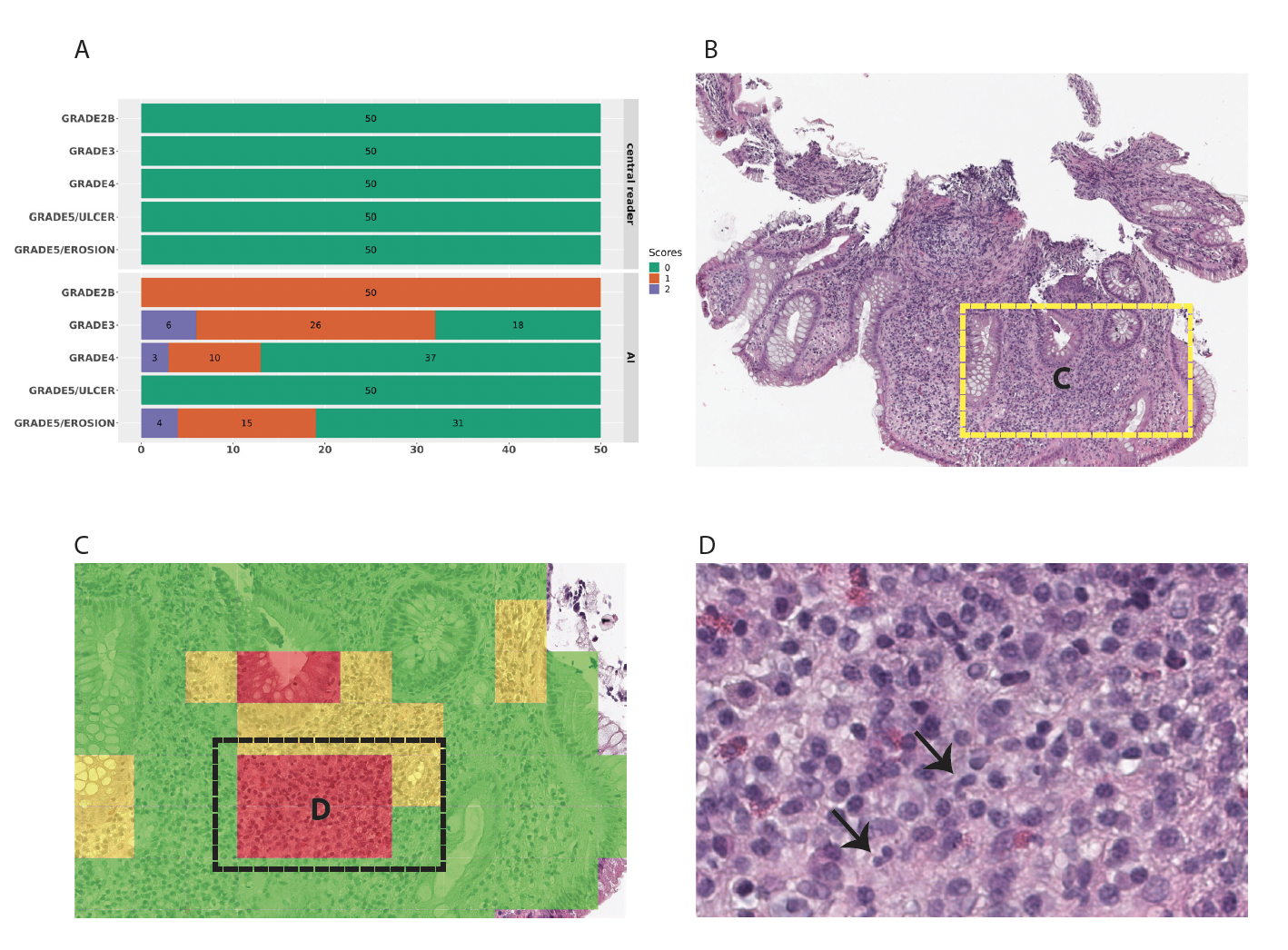
**

**Fig. S9**


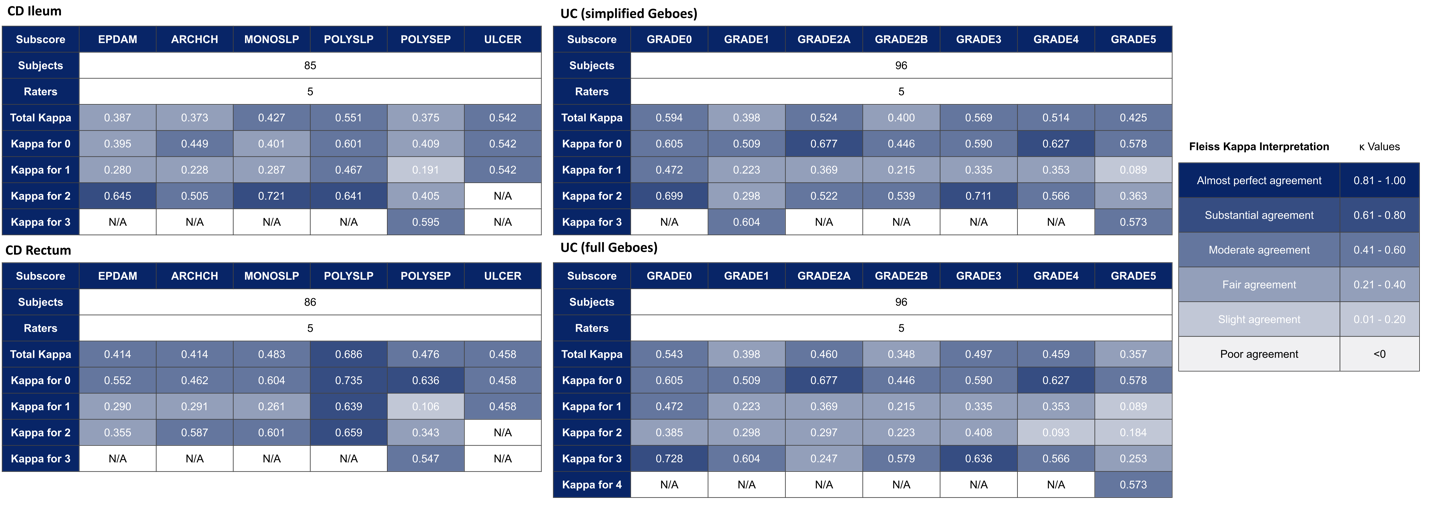


**Fig. S10**

**
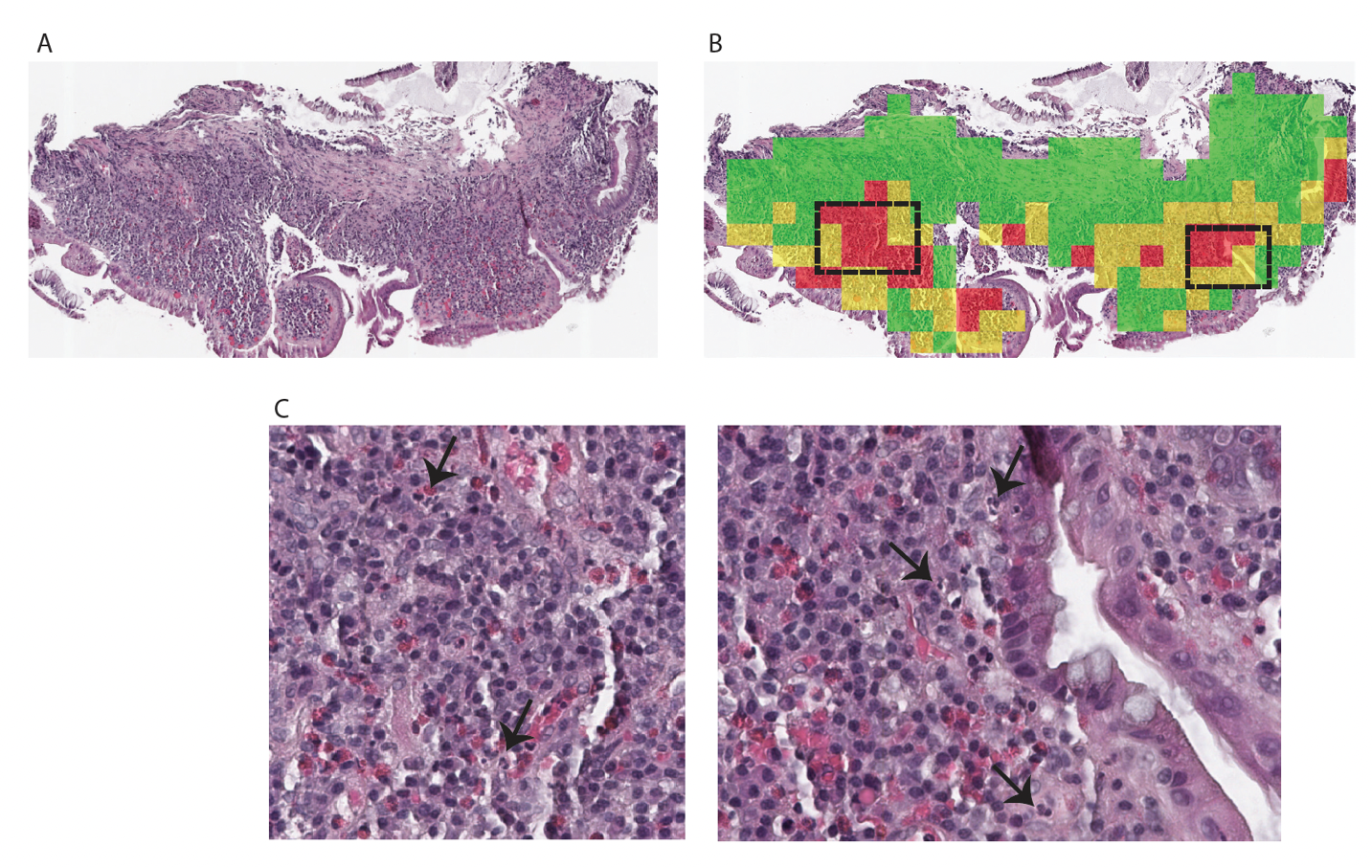
**

**Fig. S11**


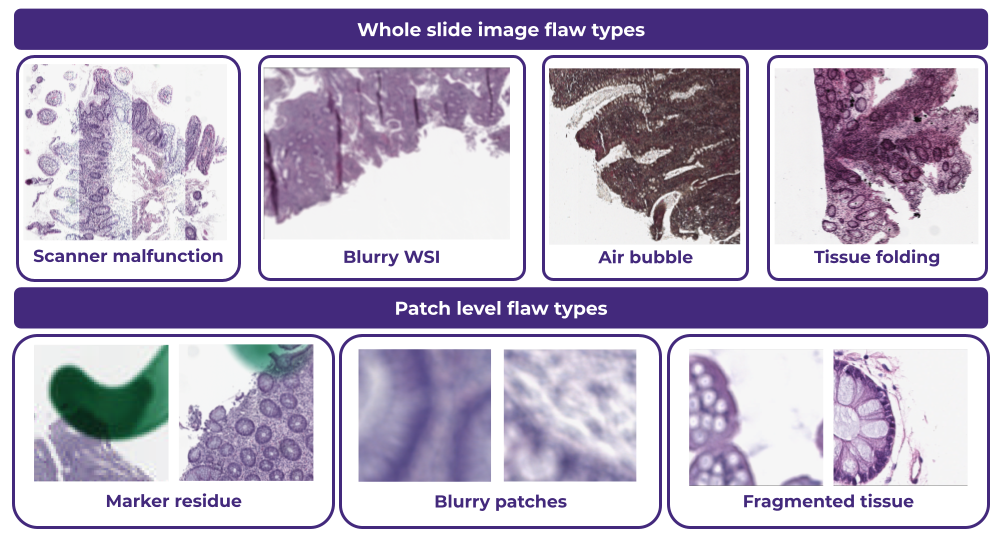


**Fig. S12**

**
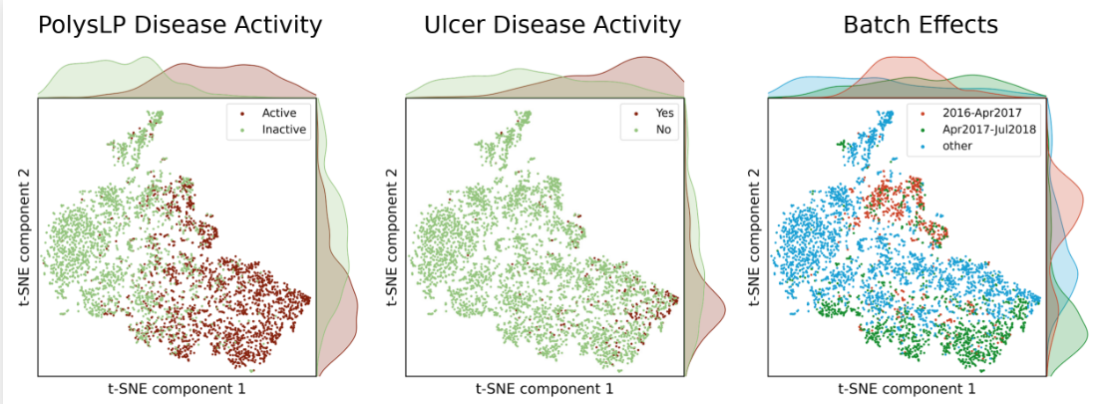
**

**Fig. S13**

**
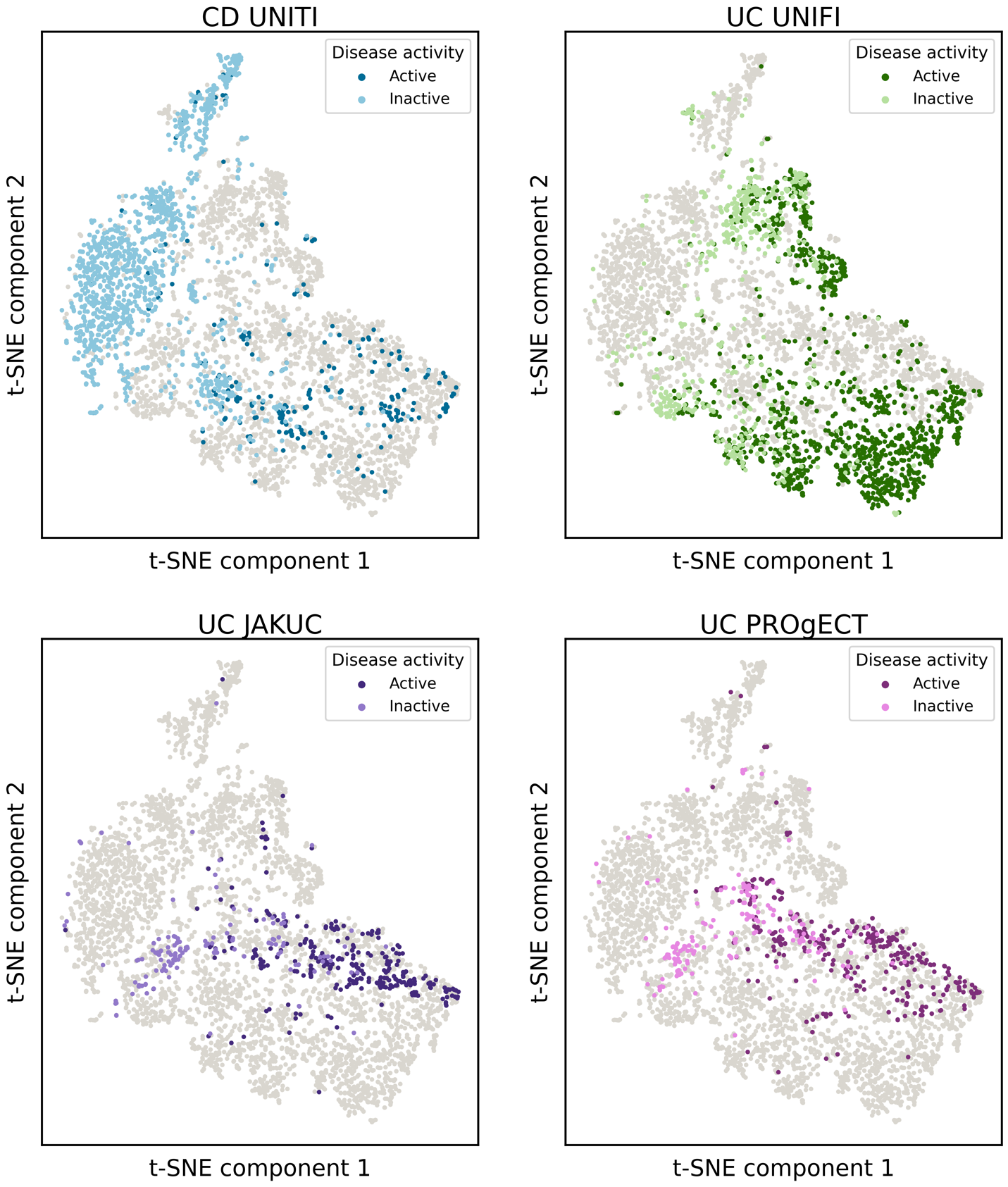
**

**Fig. S14**

**
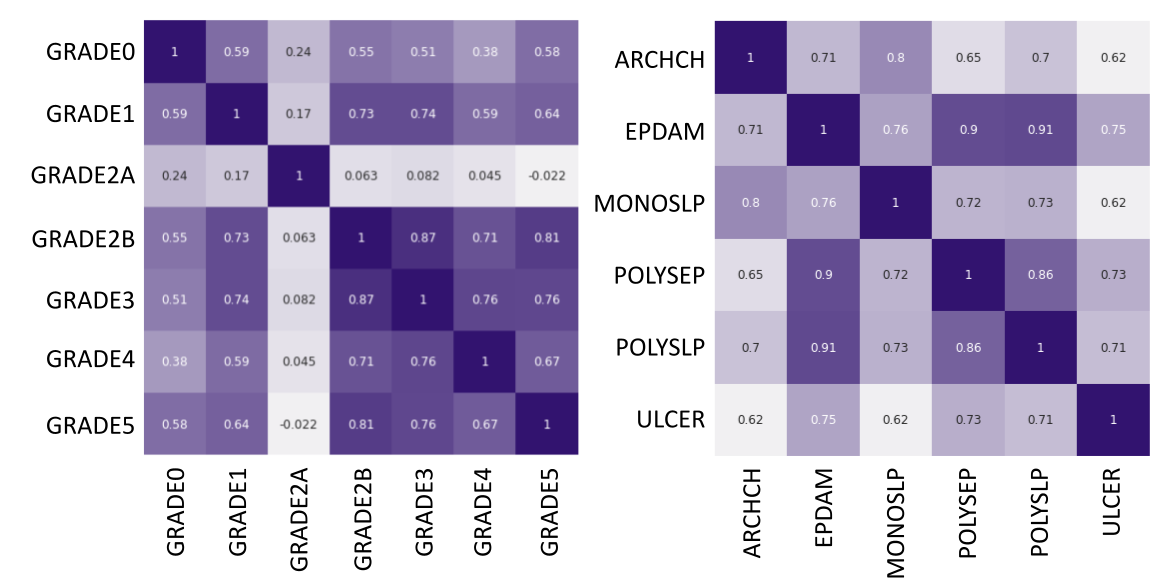
**

**Fig. S15**

**
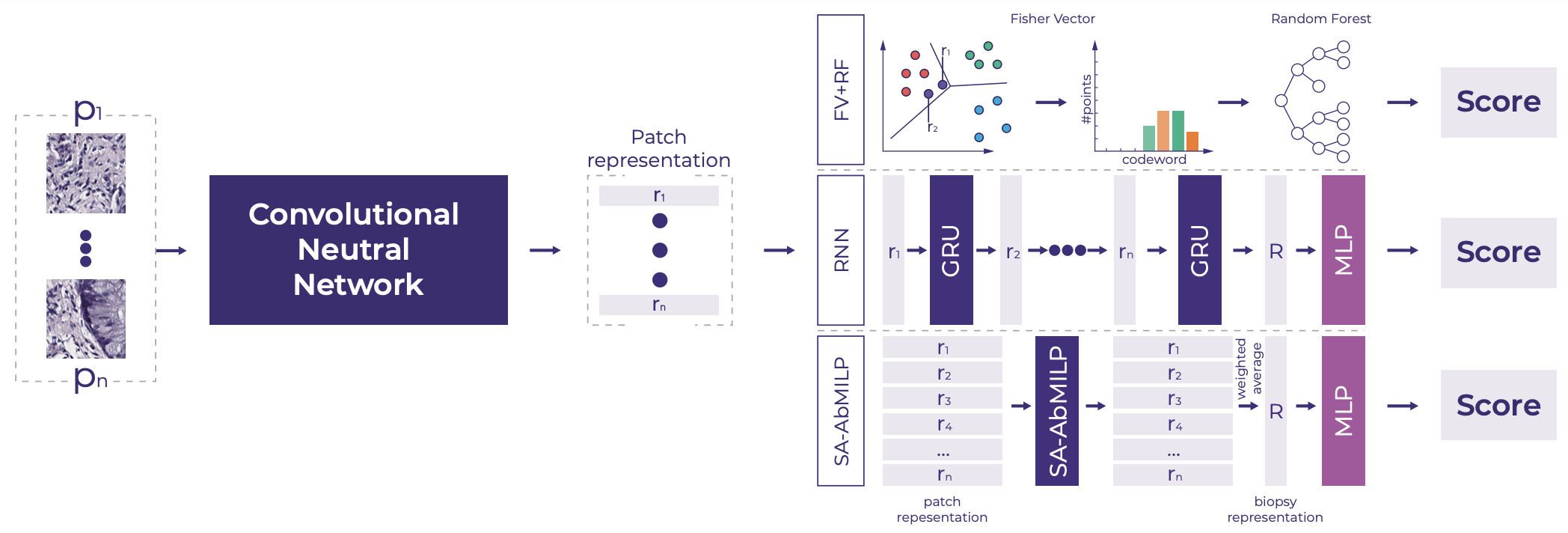
**

**Fig. S16**

**Figure S1. Global Histology Activity Score (GHAS) for Crohn’s disease as used in this study**. The presence of granuloma and number of biopsy specimens categories were not utilized [^2^](#_ENREF_2).

**Figure S2. Geboes scoring instrument for UC as used in this study.**

**Figure S3. Disease activity grades show strongly skewed score distribution.** Shown are the distributions of scores for six GHAS subgrades (top two rows; ileum and colon), Geboes subgrades (third row) and Simplified Geboes subgrades (bottom row) in the training set.

**Figure S4. A simplified Geboes scale classification was designed to address dataset imbalances that could affect model performance.** The original scores (left) that were combined are shown in bold and framed in green, with the corresponding simplified score shown on the right. Red boxes indicate scores that were not simplified but reassigned to avoid a gap in the ordinal scale. To simplify Geboes GRADE4 from a 0-3 severity scale to a 0-2 scale, we combined subgrade values 1 and 2, which represented subtle differences in severity. To accomplish this transformation, we first assessed whether tissue is normal (score = 0) or abnormal. Abnormal tissue was further classified into severity scores 1 or 2. For GRADE5 (erosions and ulcers), a 0-4 scale was simplified to a 0-3 severity scale. The presence or absence of ulcers was first determined. If ulcers were present (defined as GRADE5/ULCER), a score of 3 was assigned. For non-ulcer histological features encompassing epithelial regeneration to unequivocal erosion (GRADE5/EROSIONS), scores ranging from 1-2 were assigned. We assigned a score of 0 if ulcers or erosions were absent.

**Figure S5. A multi-stage image processing workflow was designed to predict each histological score component**. First, a Convolutional Neural Network (CNN) is trained on the image patches. Each patch used for CNN training is assigned the same disease severity label as the biopsy from which it is derived. A high-dimensional feature vector extracted from the last convolutional layer of the CNN is used as a representation of a patch. The patch representations are then aggregated into biopsy-level representations using one of three different approaches (Fisher Vector, Recurrent Neural Network (RNN), Attention Pooling). The final stage is a classifier which assigns the biopsy-level severity scores from biopsy-level representations. Two classifiers (Random Forest, Shallow Neural Network) were tested.

**Figure S6. The attention model (SA-AbMILP) outperformed the FV+RF model and showed similar performance to the RNN model in CD biopsies independent of anatomical location.** Confusion matrices are shown for each subgrade comprising the GHAS disease severity instrument for the three competing models (SA-AbMILP, FV+RF, and RNN) and were computed using the held-back testing set. The GHAS subgrades are further presented by anatomical location (colon and ileum). Each row of the confusion matrix represents the instances assessed by the human reference (central reader), while each column represents the instances predicted by the AI model. The size of each instance class is shown as a proportion of the central reader assessments, and, in parentheses, as an absolute count. Accuracy and kappa values are shown for each confusion matrix.

**Figure S7. The attention model (SA-AbMILP) showed similar performance to the RNN model in UC biopsies.** Confusion matrices computed using the held-back testing set are shown for each subgrade comprising the Simplified Geboes classification scheme for the SA-AbMILP and RNN models. Each row of the confusion matrix represents the instances assessed by the human reference (central reader), while each column represents the instances predicted by the AI model. The size of each instance class is shown as a proportion of the central reader assessments, and, in parentheses, as an absolute count. Accuracy and kappa values are shown for each confusion matrix.

**Figure S8. Representative example of AI false negative assignments of neutrophils in the lamina propria and epithelium.** The presence or absence of neutrophils in the lamina propria in this analysis of a UC sample is based upon Geboes GRADE2B score (0 - absent, 1 or greater – present), as assigned by the central reader. Shown are the distributions **(A)** of disease severity assignments for the biopsies where the AI and the central reader assignments for GRADE2B disagree, with the reference being ‘pathology present’ as assigned by the central reader. Also shown are the corresponding distributions of severity assignments for the other grades made by the central reader and AI. A representative biopsy **(B**, low magnification**)** illustrating an example where the AI makes an erroneous prediction of no neutrophils in the lamina propria **(C)** and no neutrophils in the epithelium **(D-E)** is shown.

**Figure S9. Representative example of neutrophils in the lamina propria detected by the AI model but not by the central reader.** Shown are the distributions **(A)** of disease severity assignments for the biopsies where the AI and the central reader assignments for GRADE2B disagree, with the reference being ‘pathology absent’ as assigned by the central reader. Also shown are the corresponding distributions of severity assignments for the other grades made by the central reader and AI. A representative biopsy **(B**, low magnification**)** illustrating an example where the AI identifies neutrophils in the lamina propria. Panel **C** illustrates a heatmap produced by the AI algorithm representing informative areas. Panel **D** shows a high magnification of the red patches in **C**, with neutrophils identified by arrows.

**Figure S10. Independent pathologists demonstrate moderate agreement for most subgrades as measured by Fleiss kappa.** Interrater agreement between five independent pathologists was assessed using the Fleiss kappa statistic. Total kappa reflects agreement over the entire range of possible scores for a given subgrade. For each subgrade, a separate kappa was computed for each level of disease severity (0-4, depending on the subgrade). Fleiss kappa interpretation for agreement is shown for reference.

**Figure S11. The SA-AbMILP** **model for disease activity estimation enables the identification of patches within colon biopsies that contribute to the overall score assignment.** Shown is a UC colon biopsy (A) with a superimposed heatmap of patches associated with neutrophils in the lamina propria (Geboes GRADE2B) (B); the most relevant patches for the activity assessment are colored red, intermediate patches colored yellow, and those less informative colored green. Examples of H&E stained neutrophils from the informative region are magnified in panel (C)**.**

**Figure S12. Image quality flaws may negatively influence the model training procedure.** Shown are examples of whole slide images (top) and patches (bottom) with technical flaws that were removed from consideration during model training and evaluation.

**Figure S13. The attention model (SA-AbMILP) is effective at capturing variation between the presence and absence of histopathology and also sensitive to batch effects associated with tissue preparation.** Shown are t-SNE 2D representations of biopsy-level feature vectors pooled from the model trained to classify Geboes subscore GRADE2B applied to colonic biopsies from both UC and CD clinical studies. This was done to facilitate a comparison between the presence and absence of pathology and batch effects across studies and to explore if a unified scoring instrument was feasible. Each point represents a biopsy from the training sets. The left panel shows the split between the presence (orange) and absence (green) of pathology, defined by the presence or absence of neutrophils in the lamina propria, as assessed by the central reader. The middle panel shows the split between the presence (orange) or absence (green) of ulcers, as assessed by the central reader. The right panel shows the batch effects from UNIFI study biopsies due to temporal differences in specimen processing. The points are colored based upon the change of the processing lab (red – before, green – after). Blue points correspond to biopsies from studies other than UNIFI.

**Figure S14. The variation between the presence and absence of histopathology can be captured by the attention model (SA-AbMILP) across individual clinical studies.** Shown are t-SNE 2D representations of feature vectors pooled from the network trained to classify Geboes subscore GRADE2B applied to colonic biopsies from both UC and CD clinical studies. The biopsy representations for each of the four studies comprising our training dataset are highlighted as colored points corresponding to the presence or absence of pathology, as assessed by the central reader. Biopsies shaded gray belong to the other studies and are shown for reference.

**Figure S15. Our model architecture exploits the correlations between scores of different grades within the Geboes or the GHAS scoring instruments to jointly model similar subgrades.** Shown are the heatmaps of correlations for Geboes subgrades (left panel) and GHAS subgrades (right panel) with correlation R values noted in each box. Correlations were computed based on data from the training set.

**Figure S16. Illustration of investigated AI model architectures.** All investigated models aggregate patch representation vectors to obtain a biopsy prediction. FV+RF combine Fisher Vector encoding with a Random Forest classifier. Patch representations are mapped into a space and grouped using Gaussian Mixtures. When a new biopsy is analyzed, the distance between the group centroids and the current patch’s distribution is then calculated to obtain a feature vector that is classified by Random Forest. RNN consists of GRU (gated recurrent unit) cells that process patch representation vectors one-by-one to accumulate information from all vectors and generate a biopsy representation, which is then classified by a multi-layer perceptron (MLP). SA-AbMILP uses the attention technique to analyze all patch representation vectors and assign an importance weight to them. Then, a weighted average is calculated to obtain a biopsy representation vector, which is then classified by MLP.

**References**

1. D'Haens GR, Geboes K, Peeters M*, et al.* Early lesions of recurrent crohn's disease caused by infusion of intestinal contents in excluded ileum. *Gastroenterology* 1998;**114**:262-7.

2. Li K, Friedman JR, Chan D*, et al.* Effects of ustekinumab on histologic disease activity in patients with crohn’s disease. *Gastroenterology* 2019;**157**:1019-31. e7.

3. Geboes K, Riddell R, Öst A*, et al.* A reproducible grading scale for histological assessment of inflammation in ulcerative colitis. *Gut* 2000;**47**:404-9.

4. Otsu N. A threshold selection method from gray-level histograms. *IEEE transactions on systems, man, and cybernetics* 1979;**9**:62-6.

5. Shrivastava A, Adorno W, Sharma Y*, et al.* Self-attentive adversarial stain normalization. In: International Conference on Pattern Recognition, 2021.

6. Hearst MA, Dumais ST, Osuna E, Platt J, Scholkopf B. Support vector machines. *IEEE Intelligent Systems and their applications* 1998;**13**:18-28.

7. Dalal N, Triggs B. Histograms of oriented gradients for human detection. In: 2005 IEEE computer society conference on computer vision and pattern recognition (CVPR'05), 2005.

8. He K, Zhang X, Ren S, Sun J. Deep residual learning for image recognition. In: Proceedings of the IEEE conference on computer vision and pattern recognition, 2016.

9. Deng J, Dong W, Socher R*, et al.* Imagenet: A large-scale hierarchical image database. In: 2009 IEEE conference on computer vision and pattern recognition, 2009.

10. Arpit D, Jastrzębski S, Ballas N*, et al.* A closer look at memorization in deep networks. In: International conference on machine learning, 2017.

11. Kingma DP, Ba J. Adam: A method for stochastic optimization. *arXiv preprint arXiv:14126980* 2014.

12. Ruder S. An overview of multi-task learning in deep neural networks. *arXiv preprint arXiv:170605098* 2017.

13. Zieliński B, Sroka-Oleksiak A, Rymarczyk D, Piekarczyk A, Brzychczy-Włoch M. Deep learning approach to describe and classify fungi microscopic images. *PloS one* 2020;**15**:e0234806.

14. Hochreiter S, Schmidhuber J. Long short-term memory. *Neural computation* 1997;**9**:1735-80.

15. Rymarczyk D, Borowa A, Tabor J, Zielinski B. Kernel self-attention for weakly-supervised image classification using deep multiple instance learning. In: Proceedings of the IEEE/CVF Winter Conference on Applications of Computer Vision, 2021.

16. Lin T-Y, Goyal P, Girshick R, He K, Dollár P. Focal loss for dense object detection. In: Proceedings of the IEEE international conference on computer vision, 2017.

17. Cao K, Wei C, Gaidon A, Arechiga N, Ma T. Learning imbalanced datasets with label-distribution-aware margin loss. *Advances in neural information processing systems* 2019;**32**.

18. Ba JL, Kiros JR, Hinton GE. Layer normalization. *arXiv preprint arXiv:160706450* 2016.

19. Van der Maaten L, Hinton G. Visualizing data using t-sne. *Journal of machine learning research* 2008;**9**.

20. Cohen J. A coefficient of agreement for nominal scales. *Educational and psychological measurement* 1960;**20**:37-46.

21. Li K, Marano C, Zhang H*, et al.* Relationship between combined histologic and endoscopic endpoints and efficacy of ustekinumab treatment in patients with ulcerative colitis. *Gastroenterology* 2020;**159**:2052-64.

22. Schroeder KW, Tremaine WJ, Ilstrup DM. Coated oral 5-aminosalicylic acid therapy for mildly to moderately active ulcerative colitis. *New England Journal of Medicine* 1987;**317**:1625-9.

1. https://github.com/menpo/cyvlfeat [↑](#footnote-ref-2)
2. https://scikit-learn.org/stable/ [↑](#footnote-ref-3)
3. https://pytorch.org/ [↑](#footnote-ref-4)
